# Advances in Solid-State Transformations of Coordination Bonds: From the Ball Mill to the Aging Chamber

**DOI:** 10.3390/molecules22010144

**Published:** 2017-01-17

**Authors:** Cristina Mottillo, Tomislav Friščić

**Affiliations:** Department of Chemistry, McGill University, 801 Sherbrooke Street West, Montreal, QC H1P 1W1, Canada

**Keywords:** mechanochemistry, coordination polymers, metal-organic frameworks, green chemistry, solid-state chemistry, solvent-free synthesis, reaction mechanisms, sustainable synthesis

## Abstract

Controlling the formation of coordination bonds is pivotal to the development of a plethora of functional metal-organic materials, ranging from coordination polymers, metal-organic frameworks (MOFs) to metallodrugs. The interest in and commercialization of such materials has created a need for more efficient, environmentally-friendly routes for making coordination bonds. Solid-state coordination chemistry is a versatile greener alternative to conventional synthesis, offering quantitative yields, enhanced stoichiometric and topological selectivity, access to a wider range of precursors, as well as to molecules and materials not readily accessible in solution or solvothermally. With a focus on mechanochemical, thermochemical and “accelerated aging” approaches to coordination polymers, including pharmaceutically-relevant materials and microporous MOFs, this review highlights the recent advances in solid-state coordination chemistry and techniques for understanding the underlying reaction mechanisms.

## 1. Introduction

This review provides a brief survey of recent advances in solvent-free synthesis and transformations of molecules and materials based on metal-ligand coordination bonds. The focus of the article is on metal-organic structures and it builds on previous reviews provided a decade ago by the James group [1] and the Braga group [2]. Considering that coordination chemistry in the solid state is a rapidly advancing area, as well as that a number of recent reviews have already addressed solid-state and mechanochemical transformations of inorganic [3], organic [4,5,6,7,8] and organometallic [9] compounds, this review is not intended to be comprehensive. Instead, it aims to provide an overview of the current research landscape, and takes the opportunity to highlight basic aspects of the techniques and underlying mechanisms. In doing so, we focus largely on mechanochemistry [10], but also address other emergent techniques, such as vapour-assisted reactivity, as well as thermally- and microwave-induced transformations leading to coordination polymers and MOFs.

## 2. The Role of Solvent-Free Synthesis in Coordination Chemistry

The development of solvent-free synthesis has been driven largely by the need for synthetic procedures that are cleaner and more energy- and materials-efficient than existing, solvent-based ones. This rationale is obvious in the context of organic synthesis, especially of pharmaceutically relevant molecules and materials. Until recently, a lack of established, commercially relevant targets has meant that a similar impetus for the development of solvent-free synthesis has largely been absent in the chemistry of coordination compounds. As a result, the development of solvent-free, solid-state strategies for transformations of coordination bonds has been mostly driven by academic curiosity. However, recent commercialization [11,12] of coordination polymers with open framework structures, also known as metal-organic frameworks (MOFs) [13,14,15,16,17] or porous coordination polymers (PCPs) [18,19], now creates a need for coordination chemistry with low environmental impact, high atom and energy efficiency, as well as potential for efficient, high-throughput materials screening.

**Figure 1:**
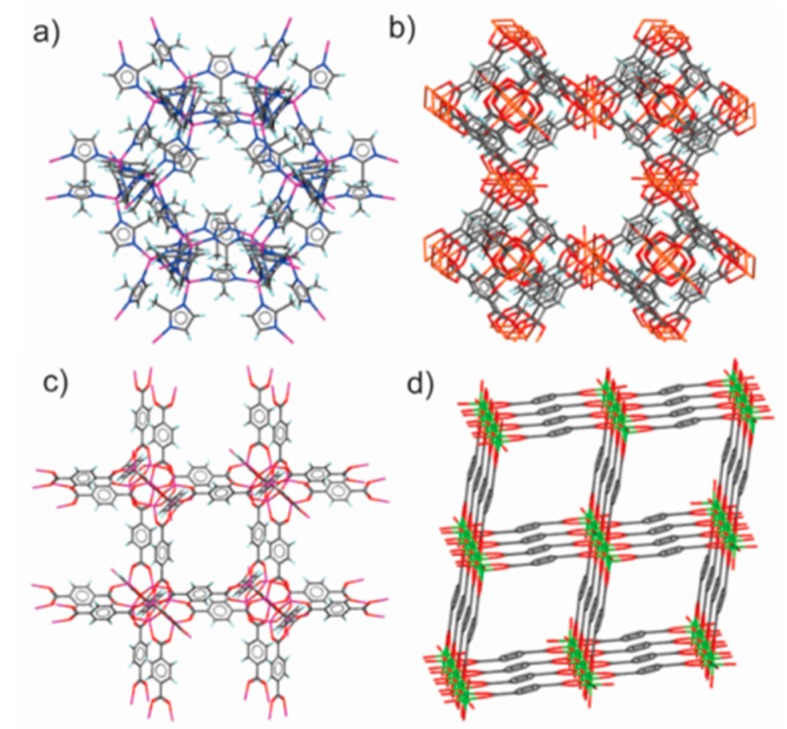
Structures of selected commercially relevant MOFs: (**a**) ZIF-8, sold as Basolite Z1200^®^ and MTA2^®^; (**b**) HKUST-1, sold as Basolite C300^®^ and MTA4^®^; (**c**) MOF-5, produced industrially by BASF; and (**d**) MIL-53, sold as Basolite A100^®^.

As a rapidly expanding family of 2- or 3-dimensional (2-D or 3-D, respectively) coordination polymers with open or even microporous architectures, MOFs have been at the forefront of materials chemistry for over two decades. Mostly based on designs involving metal centres as nodes and designer organic molecules as linkers [20,21], MOFs can access remarkable porosity and surface areas [22,23], exhibit high stability to temperature and diverse environments [24,25], and can be chemically modified [26,27,28], making them ideal contenders for diverse advanced applications [29,30,31]. The judicious combination of metal nodes and rigid organic linkers, central to the node-and-linker approach and the associated isoreticular synthetic strategy [20,32,33,34], has enabled the discovery and formation of hundreds, if not thousands, of MOFs. Of these, several have already entered commercialisation, including the highly studied ZIF-8 (also known as MAF-4, sold as Basolite Z1200^®^ or MTA2^®^) [35], HKUST-1 [36], MIL-53 [37], MOF-5 [38], and UiO-66 [39] (Figure 1).

Commercialization and unabated growth of interest in new and advanced applications of MOFs (e.g., conductivity, photovoltaic behavior, catalysis) [29,40,41] have brought into the limelight the persistent challenges of conventional, solution-based coordination polymer syntheses. These are normally solvothermal, i.e., involve high-boiling solvents (an example is *N*,*N*-dimethylformamide (DMF), which is also a teratogen), excess reagents, and often produce products in limited yields and phase purity [38,42,43]. Such limitations create strong driving forces to search for more energy-, materials-, cost-, and time-efficient processes [43,44]. The assessment that solvent accounts for up to 90% of weight used in all pharmaceutical or fine chemical batch processes [45] makes solvent-free or solvent-reduced synthesis, such as mechanochemistry [10,46,47,48] accelerated aging [49,50] and thermally-assisted [51] reactions central in developing alternative synthetic techniques. While the aim of this review is to highlight the most recent advances in solid-state coordination chemistry, more comprehensive overviews of techniques for coordination polymer synthesis can be found in recent literature [52,53,54].

Although the ability to induce transformations of solids by grinding or milling has been known for millennia [55], only recently has it been validated as a versatile method to conduct organic [56,57], inorganic [58], and organometallic [59,60] transformations leading to diverse high-value materials, including pharmaceuticals [61,62], nanoparticles [63,64], hybrid [65] and microporous materials [66,67,68]. Solid-state chemistry appears particularly amenable to the self-assembly of complex structures, through hydrogen bonding, reversible covalent chemistry or formation of coordination bonds, with a large array of literature examples demonstrating the synthesis of small molecule complexes [1,69], (supra)molecular or metal-organic clusters and cages [70,71,72], as well as coordination polymers [73,74,75]. Inspired by such work, several pioneering reports have demonstrated that not only is the solid-state synthesis of MOFs feasible, but that it can be faster and higher yielding than many alternatives [76]. Indeed, modern approaches to mechanochemistry have now made accessible almost all major MOF families, including zeolitic imidazolate frameworks (ZIFs), pillared MOFs, isoreticular materials (IRMOFs) including MOF-5, MIL-type structures and zirconium-based UiO-66 and derivatives. Moreover, solid-state synthesis in the form of mechanochemistry is also readily scalable and adaptable to continuous manufacture, e.g., by twin screw extrusion (TSE) [77,78]. Importantly, concepts fundamental to MOF synthesis in solution (i.e., the node-and-linker design, structure templating ubiquitous for the synthesis of zeolitic [79] or other types of open frameworks [80]) not only remain applicable in the solid state, but can also be leveraged for the discovery of new structures and polymorphs.

Of all solid-state techniques, mechanochemistry is becoming the focal point of efforts to design solid-state syntheses of coordination polymers and MOFs. Often found to lead to shorter reaction times and higher yields than its solution counterparts, mechanochemistry also enables the use of otherwise inert, poorly soluble but industrially highly desirable feedstocks, such as metal oxides or carbonates. The efficiency and synthetic scope of mechanochemistry are increased through modified techniques of liquid-assisted grinding (LAG, also known as solvent-drop grinding or kneading) [81,82,83,84] and ion- and liquid-assisted grinding (ILAG) [84], each involving the use of a catalytic liquid additive, or a combination of a liquid and a salt additive, respectively, as a means to induce, enhance or direct reactivity of solid reactants. A wide array of tools is available for characterization of mechanochemically obtained products, including infrared [85] and Raman spectroscopy [86], solid-state nuclear magnetic resonance (ssNMR) spectroscopy [87], terahertz spectroscopy [88], Mössbauer spectroscopy [89] and different types of electron microscopy [90]. However, due to usually small crystallite size (ranging from hundreds of nanometers to micrometer size), mechanochemically-obtained materials usually cannot be characterized directly by single crystal X-ray diffraction. Nevertheless, advances in laboratory powder diffraction equipment and software are now making structure determination from X-ray powder diffraction data an increasingly viable option for direct structural characterization of products of mechanochemistry [91,92,93,94]. The success of mechanochemistry in coordination polymer synthesis has also inspired the emergence of other synthetic approaches that dismiss the use of bulk solvents, based on vapour-assisted reactions [49,50], reactions in the melt [51], as well as microwave-assisted solid-state synthesis [95,96]. With efficiency, including high yields and low cost, of solid-state MOF synthesis becoming increasingly attractive, it is also becoming essential to determine factors, reaction kinetics, and mechanisms controlling the assembly and stability of materials under such conditions. This led to exciting advances in reaction monitoring, either ex situ (i.e., in a stepwise manner) or in situ, via real-time X-ray diffraction or Raman spectroscopy, which will also be covered in this review [97,98]. For a more detailed overview of mechanistic studies pertinent to mechanochemical transformations, we recommend recent reviews [99,100].

## 3. Mechanochemical Synthesis of Coordination Polymers

### 3.1. Mechanochemistry, History, Scope and Benefits

There is a number of methods through which mechanical force can achieve or facilitate molecular-level transformations, such as bond cleavage or formation, amorphisation and triboelectric or charge separation effects [101,102,103], including ultrasonic irradiation [104], individual molecule stretching using an atomic force microscope (AFM) [105], surface rubbing, shearing, and bulk grinding or milling. The focus of this review is on reactions by milling or grinding, which is the form of mechanochemistry most commonly applied to bulk synthesis. According to Takács [106], written records on using mechanical force to induce chemical reactions date at least as far back as 4th century B.C., when Theophrastus of Eresos described the formation of elemental mercury by manual grinding of HgS in a copper (or bronze) mortar. The small amount of vinegar used to “lubricate” this reaction brings about an uncanny resemblance to modern liquid-assisted techniques of LAG and ILAG [84]. Other, more recent examples include the formation of silver amalgam by trituration of AgCl with HgCl_2_ [107], and numerous examples of large-scale processing of slightly soluble inorganic minerals [108]. Mechanochemistry and milling are used in the synthesis of a wide range of inorganic materials, as in oxide, sulfide, carbide, nitride or boride formation [109,110,111,112,113], and are central to mechanical alloying [114,115]. Milling and grinding appear to have been adopted as synthetic techniques first by organic chemists, including Wöhler in the 19th century [116] and a number of solid-state organic chemistry research groups in the 20th century, notably Paul and Curtin [117], and Etter [118,119]. However, it is only in the past 30 years that mechanochemistry has been recognised as a reliable synthetic tool, largely by synthetic chemists interested in “soft materials”, such as crystalline and amorphous organic solids [120], including polymorphs, pharmaceutical cocrystals [121], salts [122] and coordination polymers [123,124,125,126,127,128,129]. More recently, mechanochemistry has also become of interest to researchers in metal-catalysed and organocatalytic covalent bond formation [130,131,132], including C-H activation, as well as to organometallic chemists [133,134,135]. Modern mechanochemistry has, to a large extent, been inspired by the work of Toda’s group [136,137], who demonstrated that organic reactions and host-guest inclusion can not only take place mechanochemically without solvent, but also that they can be highly specific and yield products inaccessible by conventional (i.e., in solution) means [138]. The benefits of mechanochemistry in synthetic chemistry are two-fold. Firstly, mechanochemistry can make chemical processes more environmentally-friendly by virtually eliminating solvent use, enabling reactivity at ambient conditions of temperature and pressure, shortening reaction times, and increasing yields. Secondly, there is a growing body of evidence that mechanochemical synthesis can enable the formation of products not attainable traditionally, for example by making accessible new targets or new synthetic routes which may be hindered or impossible in solution by solvent-based interferences, such as solvation effects, solubility limitations or solvolysis [139,140,141,142,143]. Historically, the simplest method to conduct mechanochemistry is with a mortar and pestle. It is our opinion, however, that the simplicity of this technique is often more than offset by inherent drawbacks: the impracticality for employing long reaction times, limited reproducibility caused by poor reporting of experimental variables (which does not include only the choice of experimenter, but also temperature, humidity, pressure, material and radius of curvature of the mortar, etc.) and difficulty of scaling-up.

**Figure 2:**
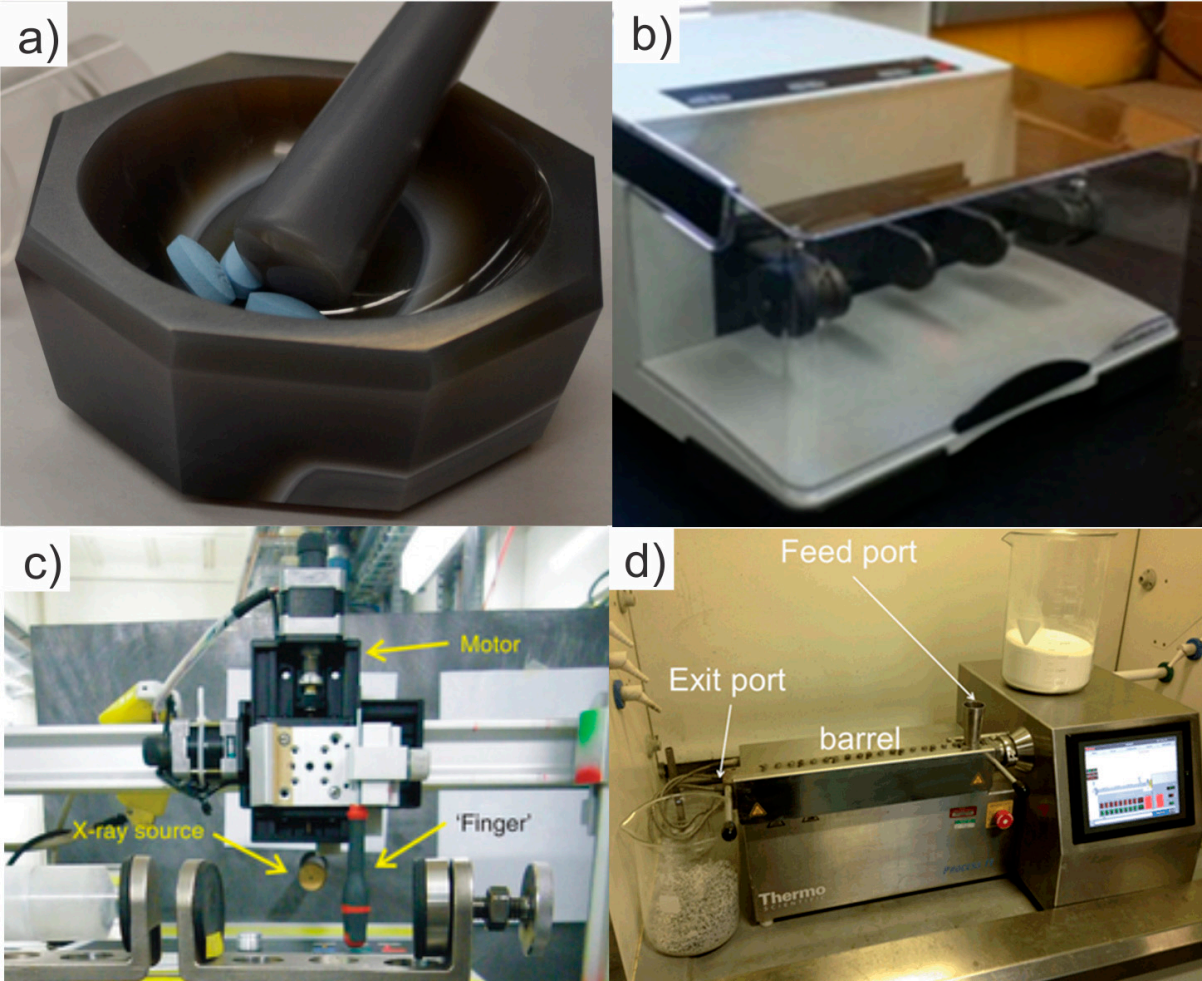
Apparatus for conducting and analyzing mechanochemical reactions: (**a**) agate mortar and pestle used for manual grinding; (**b**) automated laboratory shaker mill; (**c**) setup for in situ monitoring of mechanochemical reactions by synchrotron powder X-ray diffraction (PXRD, adapted with permission from [97]); (**d**) large-scale twin screw extrusion (TSE) reactor setup (adapted with permission from [77]).

These drawbacks are readily eliminated by using automated, electronically controlled ball mills, leading to greater reproducibility, improved control over and definition of reaction parameters (e.g., time, frequency of shaking or rotation, ratio of weight of milling media to that of sample, ratio of volume of added liquid to solid being ground, etc.), making the screening of reactivity more effective and systematic. Varying designs of milling apparatus, such as shaker mills, planetary mills [144], and twin screw extrusion (TSE) equipment (Figure 2) [77,78,145,146], can provide an array of grinding force types and scaling-up opportunities. Selection of the number and material of milling media, varying in size, number, hardness and density provides an effective and powerful way to modulate mechanochemical reaction conditions. Usually employed milling media are made of stainless steel (density 7.7 g·cm^−3^) or zirconia (density 4.5 g·cm^−3^), although alumina (density 3.95 g·cm^−3^), agate (density 2.6 g·cm^−3^) and tungsten carbide (density 15.63 g·cm^−3^) are also often employed. In addition to well-established milling technologies involving shaker (or mixer) mills (Figure 2), planetary mills or attritors, alternative setups permitting control over photochemical or thermochemical behaviour have been recently reported, such as vortex [147] and lysis mills [148], respectively.

**Figure 3:**
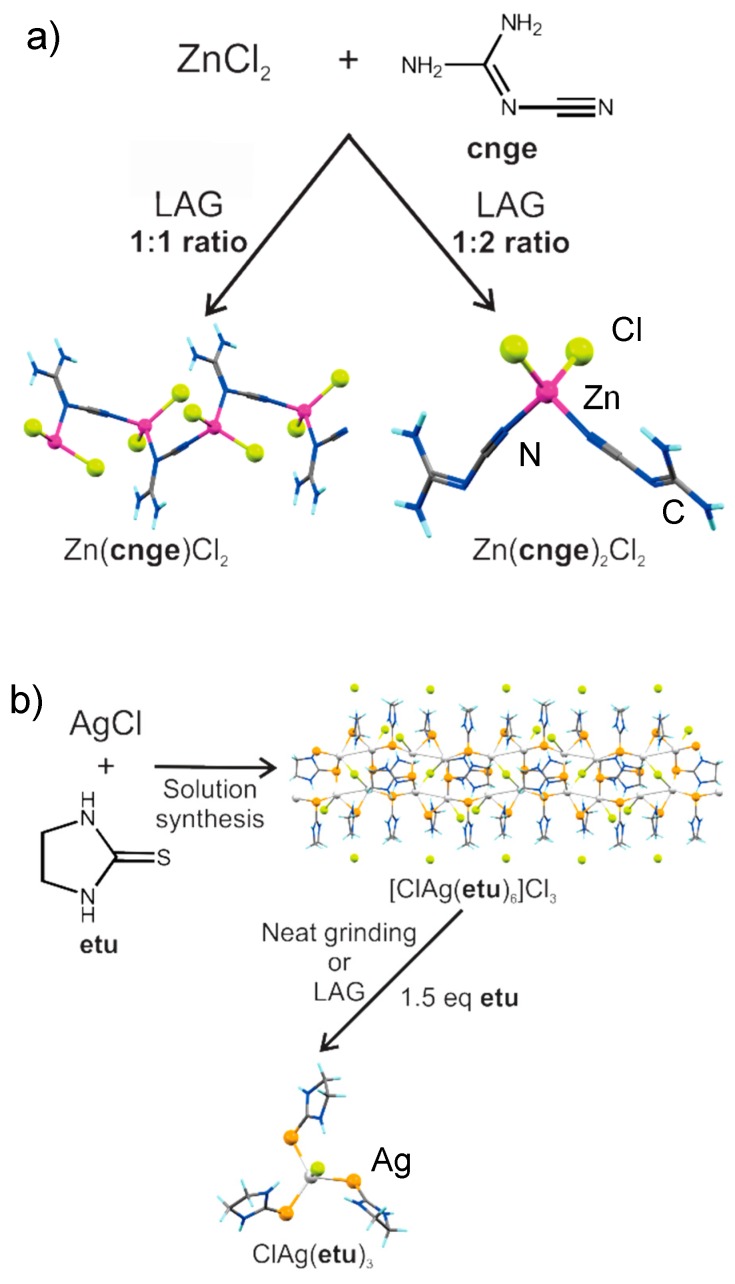
(**a**) The stoichiometry and structure of mechanochemically synthesized complexes and coordination polymers of zinc chloride and **cnge** are readily controlled by the ratio of starting materials [150]; (**b**) Mechanochemical synthesis of silver(I) complexes with ethylenethiourea (**etu**) can lead to products not obtained from solution [155].

Whereas early reports of solid-state coordination bond formation have been outlined in a pioneering review by the James group, our overview is focused on solid-state routes for the synthesis of complex metal-organic structures with extended structures, notably coordination polymers and networks [1]. One of the first examples of complex metal-organic structures formed mechanochemically was given by the Otera group, who reported the coordination-driven self-assembly of platinum(II)- and palladium(II)-based molecular squares and cages by manually grinding the respective ethylenediamine nitrate salts with bridging ligands 4,4-bipyridine (**bipy**) or tris(4-pyridyl)triazine (**tpt**), respectively [149]. Earlier solution syntheses required several weeks at 100 °C for near completion, while mechanosynthesis afforded these supramolecular structures within 10 min, in quantitative yield. Quantitative formation of products in a short time (e.g., 30–60 min) is often encountered and, in our experience, may be a hallmark of mechanochemical formation of coordination bonds, making this non-conventional synthetic approach much simpler compared to its solution counterparts, as it avoids the need for purification or filtration procedures. One recently recognized emergent benefit of mechanochemical reactivity is the excellent stoichiometric control of product formation. For example, using mechanochemical synthesis of metal complexes with cyanoguanidine (**cnge**) as a model reaction, our group has demonstrated that the stoichiometry and, therefore, topological properties of the reaction product can be predetermined by the ratio of reactants (Figure 3a) [150]. Specifically, milling of equimolar amounts of ZnCl_2_ and **cnge** selectively afforded the one-dimensional (1-D) polymer Zn(**cnge**)Cl_2_, whereas a mixture of the two reactants in the respective 1:2 stoichiometric ratio yielded selectively the discrete (0-D) complex Zn(**cnge**)_2_Cl_2_. In contrast, attempts to synthesize each complex in solution, from different solvents, always afforded mixtures of the two complexes, most likely due to differences in solubility of Zn(**cnge**)Cl_2_ and Zn(**cnge**)_2_Cl_2_. Excellent stoichiometric control was also observed for the reaction of CdCl_2_ with **cnge** [150], as well as for different examples of mechanochemical cocrystallization [151,152] and organic transformations [153,154]. Besides stoichiometric control, another highly exciting aspect of using mechanochemistry to synthesize coordination compounds is the discovery of materials that may be tantalizing to attain from solution. The Bowmaker group, for example, reported that milling of AgCl and ethylenethiourea (**etu**) in a respective 1:1.5 stoichiometric ratio resulted in the complex AgCl(**etu**)_1.5_, also obtainable from solution [155]. However, mechanochemistry also permitted the synthesis of the previously not known AgCl(**etu**)_3_, by milling of AgCl(**etu**)_1.5_ with 1.5 more equivalents of **etu** (Figure 3b). In contrast, attempts to form the hypothetical AgCl(**etu**)_2_ mechanochemically gave only mixtures of AgCl(**etu**)_3_ and AgCl(**etu**)_1.5_, thereby illustrating how mechanochemistry can be used to either screen for coordination compounds, or potentially refute their proposed existence.

### 3.2. Mechanochemical Synthesis of Coordination Polymers by Neat Grinding

**Figure 4:**
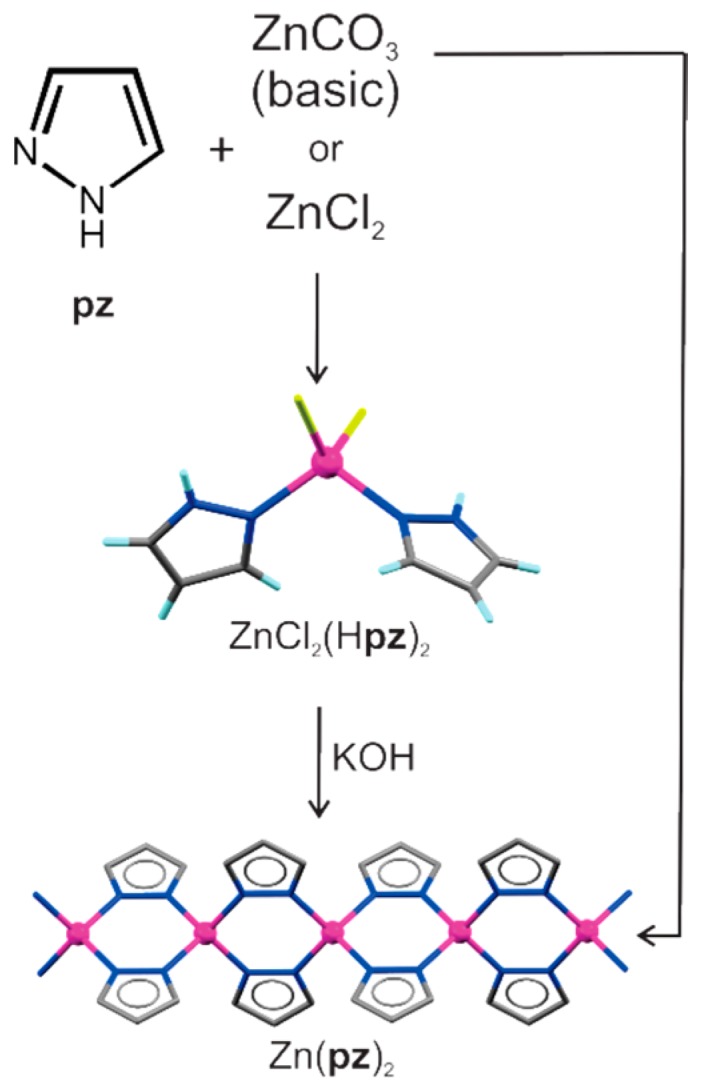
Solid-state synthesis of metal pyrazolates from metal carbonates or chlorides [164].

Mechanosynthesis by neat grinding encompasses both manual grinding of reactants, for example in a mortar with a pestle, or their milling in some type of automated milling apparatus. Thabet et al. pioneered the synthesis of coordination bonds by neat grinding, by reporting the solventless mechanochemical reaction of neutral ligands with metal halides [156]. Such ligand addition is a useful route to form complex coordination polymers. The Nassimbeni group reported the mechanochemical addition of pyrazine (**pyz**) to the one-dimensional (1-D) coordination polymer ZnBr_2_(**pyz**) [157] to yield the square-grid (*sql*) topology framework ZnBr_2_(**pyz**)_2_. This work appears to be the first report of an open metal-organic network formed mechanochemically [10]. Extensive investigations of mechanochemical formation of coordination complexes and polymers by neat grinding, as well as kneading, were reported by the Braga group. One example is the grinding reaction of silver(I) acetate (AgOAc) with trans-1,4-diaminocyclohexane (**dace**) under ambient conditions, the product of which was recrystallized in different conditions to form a series of hydrated isomeric coordination polymers [158]. Exchange of ligands or metal ions by neat grinding is also an effective route to coordination polymers [159]. This was demonstrated for the first time by the Steed group, who reported the synthesis of a 1-D coordination polymer of copper(II) acetate and 1,3-di-(4-pyridyl)propane (**dpp**), by grinding copper(II) acetate monohydrate and **dpp** in a mortar and pestle, thereby inducing the exchange of the labile water molecules coordinated onto the copper(II) paddlewheel unit for bidentate **dpp** [74]. Importantly, this transformation demonstrated the retention of the copper(II) acetate paddlewheel motif, as mechanochemical grinding effectively resulted only in the exchange of axial water ligands with bridging **dpp**. This possibility to utilize pre-formed metal-organic clusters as reactants in the formation of complex metal-organic structures was subsequently utilised in mechanochemical synthesis of the popular material MOF-5 [160]. In a report by Jobággy et al. cyanoaurate(I)-based coordination polymers were synthesized via metal exchange by neat milling of potassium cyanoaurates(I) with metal halides [161]. The method provided a more reliable pathway to the medicinally-relevant cyanoaurate(I) complexes, whose solution synthesis can result in several coexisting species which are difficult to separate. The inherent basicity of some metal salts enables the mechanochemical synthesis of coordination polymers by acid-base reaction with proton-donating ligands. For example, Kuroda et al. reported the synthesis of Co(II), Fe(II), Cu(II) and Ni(II)-based coordination polymers by milling the corresponding metal(II) acetates with the acidic 3-cyano-pentane-2,4-dione ligand (**CNacac**H) [162]. This work was followed by the mechanochemical synthesis of related Cd(**CNacac**)_2_ coordination polymers, whereby selectivity for 2-D or 3-D structures was achieved by controlling the metal-to-ligand ratio [163]. In a step towards developing a simpler and cleaner synthesis of coordination polymers via mechanochemistry by limiting the formation of solid or liquid by-products, the Orpen group inaugurated the use of basic metal carbonates to synthesize coordination polymers based on pyrazolate ligands [164]. While the carbonate ion present in the reactant provided an internal base for deprotonation of the ligand, the same materials could also be obtained from diverse metal chlorides, by performing the mechanochemical procedure in the presence of KOH as a stoichiometric external base additive (Figure 4).

### 3.3. Mechanochemical Synthesis of Coordination Polymers by Liquid-Assisted Grinding (LAG)

**Scheme 1:**
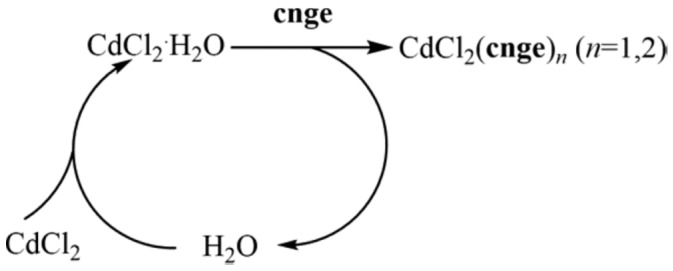
Proposed mechanism for the mechanochemical neat grinding reaction of CdCl_2_ and **cnge** facilitated by CdCl_2_·H_2_O. Adapted with permission from [167].

The above mentioned reactions mostly involve hydrated salts which are likely to release water upon grinding, or those which release other liquid by-products, such as acetic acid. It has been proposed that, in such cases, the nominally neat grinding reactions are actually enhanced by in situ generated liquid phases which aid reactivity through yet unclear mechanisms, possibly including an increase in surface mobility of reactants, their partial dissolution, or even eutectic formation [165]. A systematic screen for the reactivity of different metal salts in mechanochemical synthesis of coordination compounds, conducted by the James group, established a clear correlation between mechanochemical reaction speed and efficiency and the use of hydrated reactants and/or the formation of liquid byproducts [166]. How the water of crystallization can accelerate formally neat reactions has been shown by Užarević and co-workers [167], who performed in situ monitoring of the mechanochemical reaction of the ditopic ligand **cnge** with a mixture of anhydrous CdCl_2_ and its hydrate, CdCl_2_·H_2_O. Whereas CdCl_2_·H_2_O on its own was found to readily react with **cnge**, unlike CdCl_2_ which did not react, neat milling of the mixture of the hydrated and anhydrous form led to unexpected selective and rapid disappearance of CdCl_2_. At the same time the amount of CdCl_2_·H_2_O in the reaction mixture remained at a steady state until all of CdCl_2_ was depleted. Such behavior was explained by a mechanism in which CdCl_2_·H_2_O reacts with **cnge** first, releasing water that immediately reacts with anhydrous CdCl_2_ to regenerate the CdCl_2_·H_2_O (Scheme 1). The net outcome of such a mechanism is the observed depletion of anhydrous crystalline CdCl_2_ and **cnge**.

Such enhancement of mechanochemical reactivity can also be achieved deliberately, through the addition of small (catalytic) amounts of an external liquid additive. Specifically, the failure of neat grinding to induce reactivity in some systems can be circumvented by the addition of accelerating agents, such as a small amount of liquid. Such LAG methodology relies on a small amount of liquid to induce, facilitate, or accelerate reactivity.

**Figure 5:**
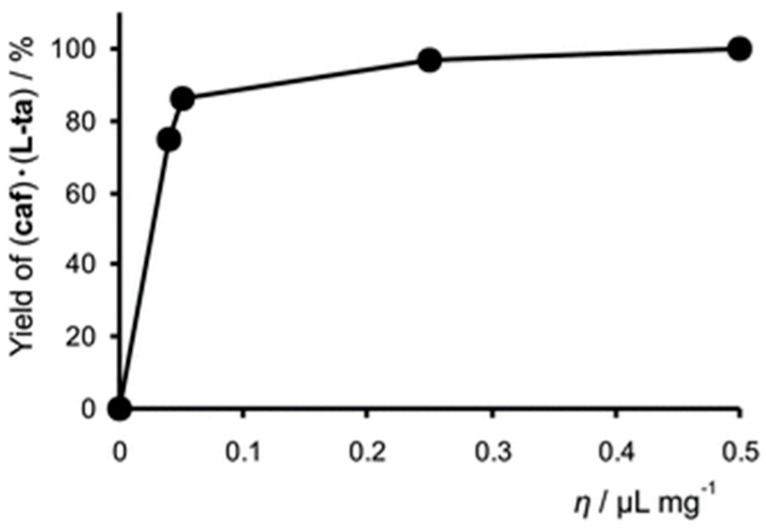
Yield of mechanochemical cocrystallization of caffeine and L-tartaric acid as a function of *η*. Adapted with permission from reference [168].

So far, there have been only a few systematic investigations on how and why the liquid additive in LAG affects the course of mechanosynthesis, all focusing on cocrystal formation. Although not dealing directly with the formation of coordination compounds, these studies are important for highlighting the aspects of LAG that distinguish it from both neat grinding and reactivity in slurries and solutions. The so far only systematic investigation of how mechanochemical reactivity is affected by the solubility of reactants in the liquid additive has focused on the formation of model pharmaceutical cocrystals involving either caffeine (**caf**) or theophylline with tartaric or malic acids as co-crystal formers [168]. This study introduced the parameter *η*, which corresponds to the ratio of added liquid (in microliters) to the weight of solid reactants (in milligrams), as a way to quantitatively describe the mechanochemical LAG environment and compare it to other types of solution- or solvent-assisted reactivity (Figure 5). This provides a convenient and quantitative way to describe LAG reactions, instead of discussing reactions with “a drop” or “a small amount” of added liquid. In such a scenario, neat grinding is defined by *η* = 0 μL·mg^−1^. The exploration of different *η* values for the same model reaction enabled LAG to be defined as the *η* range in which reactivity is not affected by low solubility of reactants (Table 1). This was empirically established to be between 0–2 μL·mg^−1^. For higher values of *η*, specifically 2–12 μL·mg^−1^, the system behaved as a slurry whose reactivity was largely affected by reactant solubility in the added liquid. For *η* > 12 μL·mg^−1^ the system was a solution with reactivity controlled by thermodynamic solubilities of reactants and products [168]. A subsequent systematic study by Hasa and co-workers revealed that changing *η* can also affect the polymorphic form of the product cocrystal [169].

Liquid-assisted grinding can provide access to synthetic pathways which are not available through neat grinding, or activate inert, insoluble starting materials such as metal carbonates. For example, the attempted synthesis of the coordination polymer [CoCl_2_(**bipy**)]_n_ by grinding anhydrous CoCl_2_ with **bipy** gave no reaction [170]. However, adding a small amount of ethanol (EtOH) to the reaction mixture resulted in rapid, full conversion into [CoCl_2_(**bipy**)]_n_ by manual grinding (kneading). In a similar manner, the solid-state reaction between Ag(**etu**)_1.5_ and 1.5 equivalents of **etu** was accelerated with the addition of a small amount of water [155]. Whereas the neat reaction did not result in full conversion after several minutes, LAG afforded quantitative yields within one minute. In addition to accelerating mechanochemical transformations, LAG has been shown to enable the solid-state and reversible self-assembly of otherwise inaccessible host-guest complexes. This was clearly demonstrated by Braga and co-workers, who investigated the effect of the liquid additive on the formation of inclusion compounds of the 1-D coordination polymer of [CuCl_2_(**dace**)]_∞_ [171]. Whereas milling of CuCl_2_ and **dace** in the presence of water as a LAG additive gave a hydrated material [CuCl_2_(**dace**)]_∞_·*n*H_2_O, doing so in the presence of a series of organic liquids such as dimethyl sulfoxide (DMSO), 2-propanol, or acetone resulted in reversible inclusion of the solvent guests into the host structure of the coordination polymer. Interestingly, the uptake of larger guest molecules, including 2-propanol or acetone, could not be achieved by the suspension of [CuCl_2_(**dace**)]_∞_ in these solvents. However, pre-kneading [CuCl_2_(**dace**)]_∞_ with a small amount of the liquid enabled formation of inclusion compounds after suspension in a series of solvents.

The lower melting points and higher solubilities of metal halides, nitrates, and acetates, make them amenable for use as reagents in solid-state synthesis [166]. However, in many respects, metal oxides, hydroxides and carbonates are ideal as precursors in coordination polymer synthesis, as they are less costly, less toxic, and give off water and CO_2_ as the only by-product. Nonetheless, the high melting points of metal oxides and their generally inert nature have, until recently, prevented their use in most solution-based or solvent-free syntheses of metal-organic compounds.

Among the first to recognize the reactivity of metal oxides towards organic ligands upon grinding was the group of Fernández-Bertran [172], who established the partial or complete formation of metal imidazolates M(**Im**)*_n_* (where M = Zn, Hg, Ag, Cd, *n* = 1, 2) by manual grinding of ZnO, HgO, Ag_2_O, and CdO with solid imidazole (H**Im**). Notably, oxides of Al, Mg, Ca, and Ba were not reactive under these conditions, suggesting that the determining step in the mechanochemical reaction was the complexation of the metal cation by the ligand, rather than deprotonation of H**Im**. Although reaction products were not characterized by any other method except infrared spectroscopy, it is very likely that the reaction involving ZnO is the first example of a mechanochemically prepared zeolitic imidazolate framework (ZIF) material. The mechanochemical reactivity of metal oxides can be greatly enhanced by the use of LAG and ILAG methodologies, as demonstrated by several reports [173,174]. For example, whereas brief (30–60 min) milling of ZnO with fumaric acid (H_2_**fum**) does not lead to the formation of a new material, milling in the presence of different liquid additives enables the rapid and quantitative assembly of structurally and chemically diverse coordination polymers. Milling of ZnO and H_2_**fum** in a 1:1 stoichiometric ratio in the presence of 3 or 4 equivalents of water selectively and quantitatively yields the previously known and structurally characterized one-dimensional coordination polymers Zn(**fum**)·4H_2_O and Zn(**fum**)·5H_2_O, respectively. Selective formation of these two coordination polymers provides another example of excellent atom efficiency and control over reaction stoichiometry achievable under milling conditions, as all components of the reaction, including the liquid additive and water by-product of the neutralization reaction, become included in the final product. Milling in the presence of catalytic amounts of methanol (MeOH) or EtOH, however, leads to the quantitative synthesis of previously unknown phases, established to be the anhydrous 3-D polymer Zn(**fum**) and a hydrated 2-D coordination polymer Zn(**fum**)·2H_2_O. The formation of different metal-organic phases by milling with different liquid additives could be readily associated to the amount and the activity of water in the liquid additive. Liquid phases of high hydrogen bonding propensity, such as MeOH or EtOH, significantly reduce the thermodynamic activity of water and facilitate the formation of anhydrous phases or materials with low level of hydration. Liquids that are not particularly efficient in reducing water activity, such as 2-propanol or acetonitrile, lead to the formation of highly hydrated materials [173,174,175]. This reactivity was readily expanded to the synthesis of 3-D open pillared MOFs by conducting the milling in the presence of a pillaring ligand, such as **bipy** or *trans*-1,2-bis(4-pyridyl)ethylene (**bpe**).

### 3.4. Mechanochemical Screening for Metal-Based Derivatives of Active Pharmaceutical Ingredients (APIs)

**Figure 6:**
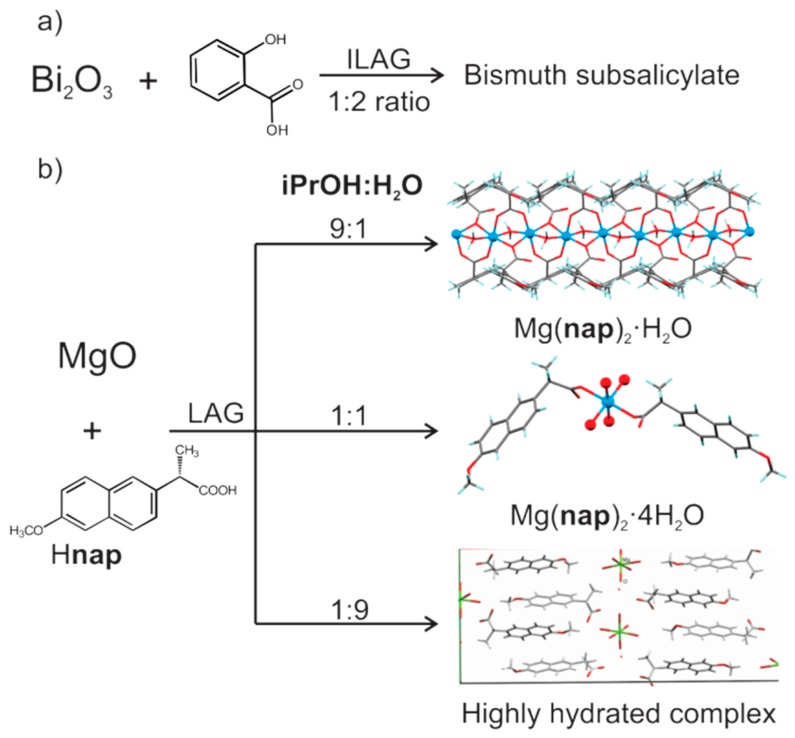
(**a**) Liquid-assisted grinding synthesis of the API bismuth subsalicylate, active ingredient in Pepto-Bismol [180]; (**b**) Increasing water activity in the LAG liquid additive leads to the formation of increasingly hydrated products in mechanochemical reaction of MgO and naproxen [184].

Mechanochemical and other types of solid-state transformations of coordination bonds (e.g., accelerated aging) are gaining popularity for the synthesis of new, metal-based derivatives of active pharmaceutical ingredients (APIs). This development comes on top of the now well-established application of liquid-assisted mechanochemistry (e.g., LAG, kneading) in screening for new API forms (polymorphs, cocrystals, solvates, salts). Although solid-state reactivity of APIs with metal-containing excipients (e.g., MgO) has been known in pharmaceutical materials science for a long time, the first targeted synthesis of metal complexes by grinding of metal salts with APIs was probably reported by the Braga and the Duarte groups, who synthesized complexes of the neuroleptic drug gabapentin by kneading of this API with zinc chloride and silver nitrate [176,177,178,179]. Subsequent work has demonstrated facile synthesis of a wide diversity of metal API complexes, including discrete complexes as well as coordination networks. Exploration of gabapentin complexation with lanthanide (La^3+^, Ce^3+^, Nd^3+^, Er^3+^) chlorides led to complexes exhibiting two modes of gabapentin coordination, in contrast to those obtained from solution. In a related study, the mechanochemical synthesis of the silver complex with 4-aminosalicylic acid led to an anhydrous product, in contrast to synthesis from solution that persistently yielded a hydrate. It can be envisaged that metal oxides, hydroxides and carbonates are highly desirable reactants for synthesis of pharmaceutical materials, not only because of their accessibility, low cost and inherently lower toxicity compared to soluble metal chlorides and nitrates, but also because their reactivity is expected to generate only water, or mixtures of water and CO_2_, as the byproduct. This was the guiding idea for developing a mechanochemical synthesis of the metallodrug bismuth(III) subsalicylate, the active component of the well-known anti-ulcer drug Pepto-Bismol, directly from Bi_2_O_3_ and salicylic acid (Figure 6a) [180]. The mechanochemical procedure, developed by André and co-workers, quantitatively yields bismuth subsalicylate by ILAG of the two reactants in a 1:2 stoichiometric ratio. However, milling of bismuth oxide and salicylic acid in a 1:4 stoichiometric ratio led to the selective formation of bismuth disalicylate monohydrate, subsequently established to be a two-dimensional coordination polymer through structure determination using high resolution X-ray powder diffraction data. The structure of bismuth disalicylate monohydrate remains the first known structure of a bismuth salicylate material without any auxiliary ligands. Finally, milling of Bi_2_O_3_ and salicylic acid in a 1:6 stoichiometric ratio yielded a bismuth trisalicylate compound. A bismuth trisalicylate was also obtained by the Andrews group by mechanochemical treatment of triphenylbismuth with salicylic acid [181]. Mechanochemical LAG reaction of MgO with either *R*- or *RS*-ibuprofen leads to the formation of a hydrated magnesium complex [182]. The complex exhibits a higher solubility in aqueous environment compared to the API itself, providing a potential explanation for higher activity of ibuprofen formulations involving MgO as the excipient [183]. In a similar fashion, LAG of MgO with salicylic acid, either alone or in the presence of additional complexation agents, led to the formation of magnesium salicylate complexes. Derivatization of APIs by complexation with magnesium ions is particularly advantageous for pharmaceutical applications because, in contrast to most other metals, magnesium is not considered to have any notable toxic effects. With that in mind, LAG with MgO was used as a means to generate new solid forms of the API naproxen (H**nap**). In particular, the systematic variation of the amount and activity of water in the milling liquid led to the formation of structurally different hydrated phases. High activity and concentration of water led to a strongly hydrated material Mg(**nap**)_2_·8H_2_O that could not be structurally characterized, but is most likely based on Mg(H_2_O)_6_^2+^ ions and **nap**^−^ counterions. In contrast, a less hydrated coordination polymer Mg(**nap**)_2_·H_2_O was obtained by LAG at lower water activity. At intermediate water activity levels an intermediate hydrate Mg(**nap**)_2_·*x*H_2_O (*x* = 3.5–3.7) was obtained, with Mg^2+^ ions octahedrally coordinated by H_2_O and **nap**^−^ ligands (Figure 6b) [184].

### 3.5. Mechanochemical Synthesis of MOFs by Neat Grinding

**Figure 7:**
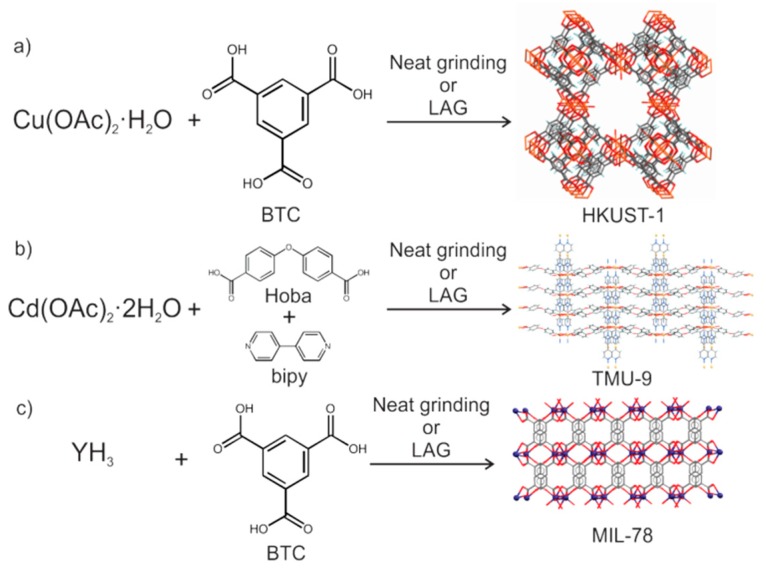
Mechanochemical syntheses of various MOFs by neat grinding or LAG: (**a**) Synthesis of HKUST-1 from a copper salt and trimesic acid [186,187,188]; (**b**) Synthesis of the pillared MOF TMU-9 from cadmium acetate [189]; (**c**) Solid-state synthesis of yttrium-based MOF MIL-78 [191].

The first entry of mechanochemistry into the synthesis of microporous MOFs was the neat milling synthesis of copper(II) isonicotinate, reported by the James group [185]. The MOF was prepared in quantitative yield by brief (10 min) milling of solid copper(II) acetate monohydrate (Cu(OAc)_2_·H_2_O) with isonicotinic acid using a shaker mill. The product was obtained in the form of a solvate, with water and/or acetic acid by-products included in the framework pores. The porous framework was obtained by evacuating the mechanochemical product at 200 °C [185]. Analogous reactivity was employed independently by the James and by the Emmerling group for the synthesis of one of the archetypal MOFs, HKUST-1, by milling Cu(OAc)_2_·H_2_O with 1,3,5-benzenetricarboxylic acid (H_3_**btc**, trimesic acid) (Figure 7a) [186,187,188]. The mechanochemically prepared MOFs have been shown to have identical or even superior properties, including microporosity and surface area, compared to those made from solution. Neat grinding was also successful in preparing MOFs based on diverse other metals and linkers. A recent example is the synthesis of a porous cadmium-based MOF from cadmium acetate, 4,4′-oxybis(benzoic acid), and **bipy**, reported by the Junk and Morsali groups (Figure 7b) [189]. Zhang et al. demonstrated the synthesis of soluble coordination polymers starting from phenol ligands and zinc acetate [190]. A so far unique example of using a metal hydride as a precursor in the mechanosynthesis of a MOF is the formation of an yttrium-based framework by neat milling of YH_3_ with H_3_**btc** (Figure 7c) [191].

Although solution-based (including solvothermal) synthesis of MOFs is highly dependent on using soluble metal salts, in particular chlorides, nitrates or acetates, such approaches have recently been criticized by manufacturing industries due to unavoidable safety risks and environmental challenges, due to inherent toxicity and corrosive nature of such salts, as well as the formation of waste mineral acids that must be neutralized through stoichiometric base, generating waste salts [192,193]. It is, therefore, not surprising that industrial researchers have recognized a need to develop alternative approaches to MOF synthesis, that would avoid solvothermal treatment and utilize safer, less corrosive but also significantly less soluble reagents, such as sulfates, oxides, hydroxides or carbonates. Several groups have been pursuing mechanochemistry for the efficient synthesis of MOFs from metal oxides or metal carbonates [194,195]. Whereas most of this work was achieved through the application of LAG and ILAG techniques, there are also examples of 3-D open MOFs obtained by neat grinding. One of the earliest reported examples is the synthesis of open MOFs from lanthanide(III) carbonates and H_3_**btc**. The mechanochemical reactions readily yielded 3-D frameworks and, by using mixtures of carbonates of different lanthanide metals, it was also possible to obtain MOFs with different metal nodes [196].

The neat grinding synthesis of MOFs is also adaptable for large-scale continuous manufacture of commercially-relevant MOFs, as demonstrated by the James group who described the synthesis of aluminium-based MOF Al(fumarate)(OH) from fumaric acid and hydrated aluminium sulfate by TSE [77]. Neat milling is not an efficient approach for the synthesis of ZIFs from a metal oxide, as reported by Beldon et al. who reported incomplete conversion of ZnO into the commercially relevant framework ZIF-8 by ball milling with 2-methylimidazole (H**MeIm**) [197]. This result was subsequently corroborated by real-time reaction monitoring using in situ synchrotron X-ray powder diffraction. Although the reaction did not reach completion within 90 min, in situ collected X-ray diffractograms clearly indicated the formation of the sodalite-topology (SOD) ZIF-8 [97,99]. More information on the mechanism of this and potentially other neat grinding transformations was provided by the Tanaka group, who used high- resolution scanning electron microscopy to establish the formation of core-shell nanostructures consisting of ZnO encapsulated by the product ZIF-8 [198].

### 3.6. Mechanochemical Amorphization of MOFs

Whereas mechanochemical treatment of MOFs under LAG or ILAG conditions generally leads to highly crystalline materials, extended neat grinding was found to induce the loss of crystallinity, leading to the formation of amorphous MOFs [199]. The formation of such metal-organic glasses by milling or through melt-quenching has been extensively studied by the Bennett and Cheetham groups [200,201], focusing largely but not exclusively [202] on the mechanochemistry of zeolitic imidazolate frameworks (ZIFs, a subset of metal azolate frameworks, or MAFs [203]). Currently, amorphous MOFs still represent a rare and novel line of materials [199], whose structural characterization requires the use of advanced techniques, such as X-ray pairwise distribution function analysis [204], solid-state NMR or positron annihilation lifetime spectroscopy [205], often coupled with theoretical modelling. It is very likely that, with the development of applications of amorphous MOFs, their synthesis will become one of the prominent, notable uses of neat mechanochemistry.

### 3.7. Mechanochemical Synthesis of MOFs Using LAG and ILAG Methodology

The addition of a small amount of liquid has been shown to dramatically enhance or accelerate the synthesis of coordination polymers from oxides or carbonates. An early demonstration of such reactivity was provided by Adams and co-workers, who demonstrated the assembly of coordination polymers by LAG or neat milling reaction of transition metal carbonates with protonated hydrochloride salts of organic linkers, such as **bipy** [206]. Variation of the liquid additive in LAG offers a means to direct the formation of metal-organic structures, as demonstrated by Yuan et al. who explored the mechanochemical reactions of basic zinc carbonate and terephthalic acid in the presence of different liquid additives [195]. Milling with water led to the formation of a 1-D coordination polymer zinc terephthalate dihydrate, whereas milling in the presence of MeOH yielded the 3-D close-packed monohydrate framework. In contrast to these close-packed materials, milling with DMF yielded an open structure with square-grid topology, consisting of two-dimensional sheets based on zinc carboxylate paddlewheel secondary building units (SBUs) interconnected by terephthalate linkers. These mechanochemically obtained products readily interconverted by LAG with an appropriate liquid. In contrast, attempts to achieve similar structural interconversions by stirring of the materials in the appropriate solvent were largely unsuccessful, indicating enhanced structural flexibility of metal-organic materials under milling conditions. In the first example of mechanochemical synthesis of open MOFs from a metal oxide, pillared MOFs were synthesized by ball milling of ZnO, H_2_**fum** and either **bipy** or **bpe** as the pillaring ligand. While neat grinding did not result in any significant conversion, LAG with DMF resulted in quantitative formation of the targeted 3-D pillared MOFs [173]. Importantly, the authors established that the reaction progress was limited by the amount of liquid present. The minimum amount of liquid required for the complete conversion of the reaction mixture was calculated from the published structures of the corresponding MOF with solvent included in the pores. If the liquid content was insufficient to completely fill the voids of the nascent MOF, the reaction would not lead to completion, highlighting a dual role of the liquid additive in MOF synthesis: as a means to accelerate the reaction, and as a space filling or even structure-templating agent. Importantly, the LAG synthesis of these pillared MOFs was readily conducted using either MeOH, EtOH or *i*-PrOH as the liquid additive, providing a means to completely avoid the use of teratogenic DMF in pillared MOF synthesis. The mechanochemically synthesized MOFs were readily desolvated by heating, as evidenced by structural characterisation of the MOF based on **bpe** pillars from PXRD data collected on the desolvated material. Similarly, the structure of the guest-free pillared MOF based on fumarate and **bpy** ligands was determined by Fujii and coworkers from PXRD data collected on thermally desolvated materials prepared by LAG from ZnO [174].

**Figure 8:**
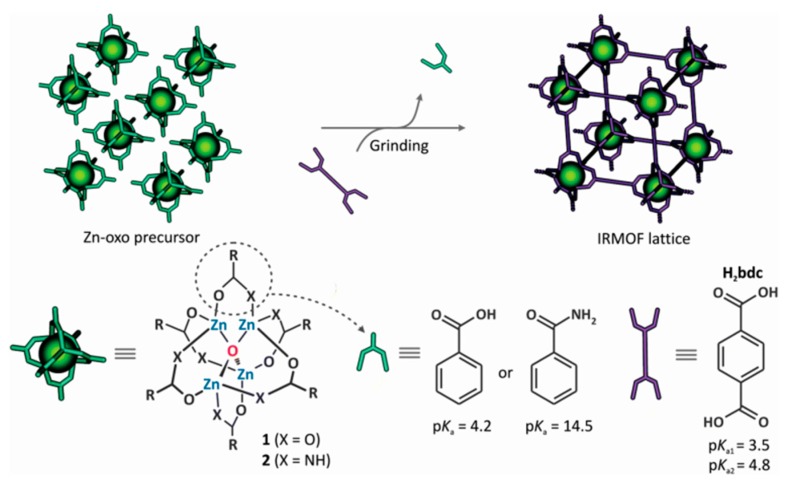
Solid-state mechanochemical synthesis of the archetypal MOF-5 material from pre-formed oxo-zinc precursors. Adapted with permission from [160].

The use of LAG was also central in developing a mechanochemical synthesis of the archetypal MOF-5 (also known as IRMOF-1) material by the Lewinski group and coworkers. The synthesis of MOF-5 was achieved by mechanochemical reaction of terephthalic acid with zinc-oxo carboxylate or amidate clusters containing the pre-assembled Zn_4_O^6+^ unit (Figure 8) [160]. Whereas the neat reaction using the benzoate-based cluster proceeded poorly in the absence of LAG additive, adding a small amount of *N*,*N*-diethylformamide resulted in the full conversion of reactants to MOF-5 which, after evacuation, exhibited a high surface area of 1831 m^2^/g. A similar strategy was applied by Užarević and co-workers [207] for the synthesis of zirconium-based UiO-66 and UiO-66-NH_2_ frameworks, based on 12-coordinated hexanuclear zirconium oxo-clusters. The MOFs were readily assembled by LAG of terephthalic acid or 2-aminoterephthalic acid with pre-assembled hexanuclear clusters coordinated with methacrylate or benzoate ligands. Importantly, however, the UiO-66 framework could readily be assembled in a multi-component one-pot process, starting from zirconium(IV) propoxide, terephthalic acid and water. In all cases, the mechanochemical reactions provided rapid and easy access to these much-sought frameworks, whose efficient synthesis in solution has so far been the target of much arduous investigation [39]. Importantly, LAG synthesis was readily conducted using methanol as the liquid additive, in that way completely displacing DMF from the synthesis, and yielded materials with high porosity and catalytic activity for hydrolytic breakdown of nerve gas analogues.

An interesting example of mechanochemical MOF synthesis was provided by the Matoga group [208], who demonstrated the mechanochemical transformation of a neutral manganese isonicotinate MOF (JUK-1) into a charged, proton-conducting MOF by neat grinding or LAG with NH_4_SCN. The mechanochemical reaction involves the attachment of isothiocyanate ligands onto and rearrangement of manganese-based MOF nodes, resulting in an anionic 2-D framework material containing NH_4_^+^ cations suitable for proton conduction. An early approach to mechanochemical ZIF synthesis was by grinding of imidazolium halometallate salts with an external base, such as KOH, which was reported by the Orpen group [209].

Whereas the available literature indicates that carbonates and hydroxides are readily applied as precursors for LAG synthesis of MOFs, the use of metal oxides is often more challenging, as demonstrated by attempted syntheses of ZIFs from ZnO that did not reach complete conversion either by neat grinding or by LAG. Reactivity of metal oxides can, however, be facilitated by ILAG, i.e., by conducting LAG in the presence of a catalytic amount of a simple salt. The first report of the ILAG technique addressed the formation of the popular pillared MOF based on open square grid sheets of zinc terephthalate separated by 1,4-diazabicyclo[2.2.2]octane (**dabco**) as the pillaring ligand [194]. Regardless of the choice of milling liquid, the pillared MOF could not be synthesized by simple LAG of ZnO, terephthalic acid and **dabco**, giving instead mixtures of **dabco** terephthalate and ZnO. This is in clear contrast to analogous pillared MOFs based on fumaric acid, which were readily obtainable by LAG. However, LAG in the presence of a catalytic amount (ca. 5 mol % with respect to Zn) of nitrate ions, in the form of an ammonium or alkaline metal nitrate, rapidly (within 30 min) yielded the expected MOF. Replacing nitrates with sulfates in this ILAG procedure yielded a different pillared MOF based on hexagonal, Kagome-topology zinc terephthalate sheets, demonstrating the active role of the salt additive in controlling the topology of the MOF. Besides leading to a topologically different product, sulfates were also significantly more active as ILAG additives, enabling MOF synthesis at amounts as low as 300 ppm with respect to zinc. The use of ILAG also enabled quantitative, room-temperature formation of close-packed as well as microporous ZIFs by milling, using only stoichiometric amounts of reagents [197]. In particular, catalytic amounts of ionic additives, such as NH_4_NO_3_, ammonium methanesulfonate (NH_4_CH_3_SO_3_) or (NH_4_)_2_SO_4_ enabled the complete conversion of ZnO into ZIFs based on H**Im**, H**MeIm** and 2-ethylimidazole (H**EtIm**). Both the liquid and the salt additives in ILAG were found to actively direct the topology of the ZIF products, which were all obtained within 30 min, at room temperature. Importantly, the ILAG approach enabled the synthesis of ZIF-8, whose typical solution-based syntheses require large amounts of solvent (e.g., DMF, or concentrated aqueous ammonia), reaction times in hours or days, and afford moderate yields [210]. The chemical and physical properties, including the surface area, of mechanochemically made ZIF-8 were identical to the conventionally prepared material.

The development of mechanochemical techniques over the past 10 years has brought forward significant advantages for the synthesis of MOFs. Although the structural characterization of mechanochemically prepared MOFs is more demanding than in the case of solvothermally-obtained samples, which can often be readily characterized through single crystal X-ray diffraction, the advantages of mechanosynthesis lie in offering a cleaner, simpler, rapid and readily controllable approach to synthesis. Importantly, mechanochemical MOF syntheses can readily be optimized (e.g., by varying the choice of liquid additive in LAG, or of the liquid and salt additives in ILAG) to achieve the quantitative formation of a topologically pure product, without resorting to stoichiometric auxiliaries or excess reagents that are often encountered in solution syntheses. A selection of respective advantages and disadvantages of different mechanochemical approaches to microporous MOFs are highlighted in Table 2, in comparison with conventional solution-based (including solvothermal) synthetic routes.

## 4. Recent Mechanistic Studies

### 4.1. Ex Situ (Stepwise) Monitoring of Mechanochemical Reactions

**Figure 9:**
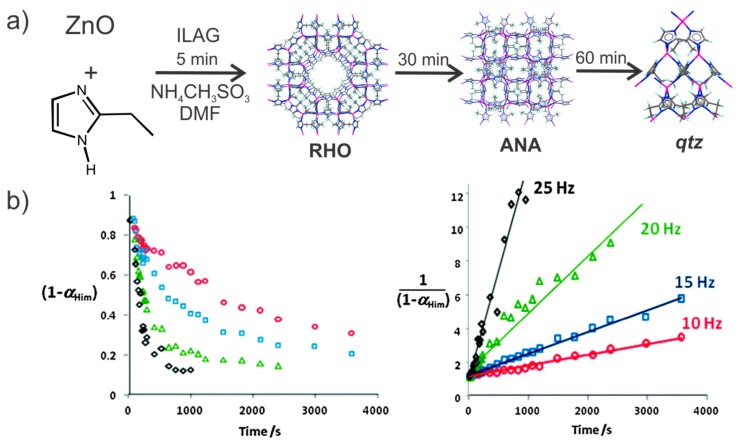
(**a**) Ex situ monitoring of the synthesis of zinc 2-ethylimidazole-based ZIFs shows progression from low-density MOFs early on in the reaction, which then collapse to more dense phases upon further milling [97,197]; (**b**) Plots of the fraction of H**Im** remaining as a function of time in the reaction between ZnO and H**Im** to form the ZIF Zn(**Im**)_2_ at milling frequencies of: 25 Hz (black), 20 Hz (green), 15 Hz (blue), and 10 Hz (red). Adapted with permission from [213].

Due to the recent rapid growth of synthetic applications of mechanochemical milling and grinding, the understanding of underlying mechanisms is becoming a highly active area of research. Until very recently, the analysis of the course of mechanochemical reactions has been possible only through stepwise, ex situ monitoring techniques, based on periodically interrupting the grinding or milling process and analyzing the reaction mixture through available solid-state analytical techniques. For this purpose, a wide range of techniques have been applied, most notably PXRD, Raman or attenuated total reflectance (ATR) infrared spectroscopy, Mössbauer spectroscopy, surface area analysis, solid-state nuclear magnetic resonance (ssNMR) spectroscopy, and thermal analysis (e.g., thermogravimetric analysis and/or differential scanning calorimetry). In many cases, structural characterization of mechanochemical products is achieved by comparing the experimental X-ray diffractogram of the powdered reaction mixture to one simulated from crystallographic data available in the Cambridge Structural Database (CSD). In certain cases, structural characterization of previously not known materials is also possible through PXRD structure solution techniques. Alternatively, single crystal X-ray diffraction is also applicable in case that mechanochemically prepared phases can be recrystallized in the form of sufficiently high quality single crystals [124]. Although mechanistic studies of mechanochemical reactions involving coordination compounds are rare, they appear to suggest general trends in reaction mechanisms. In particular, different ex situ studies of the formation of coordination polymers and MOFs by LAG and ILAG reveal stepwise mechanisms in which an initially formed low-density or highly solvated product is transformed, directly or via intermediate phases, into a denser, less solvated final product. This is well illustrated by stepwise monitoring of the reaction between ZnO and H_2_**fum** in the presence of three equivalents of water [175]. Although the final product of the reaction is the tetrahydrate coordination polymer Zn(H_2_O)_4_(**fum**), whose composition is dictated by the stoichiometric composition of the reaction mixture, interrupting the milling after five minutes reveals the intermediate formation of the more solvated pentahydrate Zn(H_2_O)_4_(**fum**)·H_2_O. Appearance of Zn(H_2_O)_4_(**fum**)·H_2_O as the reaction intermediate was explained by a mass action effect, as the relative amount of water to the nascent zinc fumarate polymer is large in early stages of the reaction. The described stepwise mechanism was subsequently confirmed in an independent study by the Boldyreva group [211]. A stepwise reaction sequence was also observed in the ball milling synthesis of copper(II) acetate monohydrate from CuO: milling of CuO with a stoichiometric amount of acetic acid, in the presence of a catalytic amount of H_2_O, initially yields an acetic acid solvate of the copper(II) acetate paddlewheel dimer. The final product of the reaction is the anticipated copper(II) acetate paddlewheel dimer. Again, the intermediate formation of a solvate was explained by the initially large amount of acetic acid reactant with respect to the nascent copper(II) acetate complex [175]. An ex situ mechanistic investigation of the mechanochemical ZIF synthesis was reported by Beldon et al. [197], who established and analyzed the ILAG reaction of ZnO and H**EtIm**. Whereas the final reaction product was the close-packed quartz (*qtz*) topology Zn(**EtIm**)_2_, as established by structure determination from PXRD data, interrupting the reaction at different time intervals revealed the initial formation of the open, low-density zeolite ρ (RHO) topology framework, RHO-Zn(**EtIm**)_2_, which is subsequently replaced by the denser analcime (ANA) topology framework ANA-Zn(**EtIm**)_2_. Upon further milling, ANA-Zn(**EtIm**)_2_ transforms into the final product, *qtz*-Zn(**EtIm**)_2_ (Figure 9a) [97,99,197]. Stepwise formation of *qtz*-Zn(E**tIm**)_2_ can again be explained by a mass action effect, as the relative amount of the liquid additive with respect to the nascent Zn(**EtIm**)_2_ coordination polymer is certainly larger in the initial moments of the reaction. However, the RHO→ANA→*qtz* sequence is also consistent with the Ostwald’s rule of stages, as it corresponds to the transformation of a framework with a low tetrahedral density (number of tetrahedral centres per 1000 Å^3^,) and, therefore, low thermodynamic stability, into structures of increasing tetrahedral density and, therefore, higher stability [212].

The first investigation of kinetics of the mechanochemical synthesis of an open MOF was reported by the James group, by using Raman spectroscopy to monitor the LAG synthesis of an open Zn(**Im**)_2_ framework in the presence of DMF [213]. The difference in characteristic Raman scattering signals of free and coordinated imidazole species enabled time-resolved observation of the ZIF formation. A plot of the time-dependent variation in the fraction of unreacted H**Im** was consistent with a 2nd-order reaction kinetics law (Figure 9b). The unexpected similarity of the mechanochemical LAG reaction kinetics to a rate law that is normally observed in dilute solutions has been explained through a pseudo-fluid model of reaction kinetics, wherein reaction progress depends on the number of collisions between the ZnO and H**Im** particles. This was buttressed by the high dependence of the reaction rate on the milling frequency which, presumably, should directly affect the frequency of contacts between reactant particles. In contrast, the reaction rate, for a set milling frequency, was not affected by changes in temperature.

One persistent problem of stepwise reaction monitoring is in ensuring a sufficiently small time interval between measurements. Highly impressive work in that context has been presented by the Emmerling group, who demonstrated the rapid stepwise analysis of the ball milling synthesis [214] of a novel bismuth coordination polymer (H_2_**Im**)(Bi(**bdc**)_2_) (**bdc** = benzene-1,4-dicarboxylate). Reaction monitoring by PXRD analysis at time intervals as short as 10 s revealed that the reaction proceeds through an intermediate Bi_6_O_5_(OH)_3_(NO_3_)_5_(H_2_O)_3_ phase, which appears 10 s into milling and disappears after one minute.

### 4.2. In Situ and Real-Time Monitoring of MOF Synthesis

Techniques for stepwise (ex situ) reaction monitoring are instrumentally simple, readily accessible in most laboratories and, as described in the previous section, can provide valuable information on the course of mechanochemical reactions. However, ex situ studies are also subject to significant limitations. Specifically, mechanochemical reactions may proceed too quickly for reliable ex situ analysis, as reaction intermediates, including crystalline and amorphous phases, can appear and disappear on the time scale of seconds. Moreover, physicochemical transformations induced by milling are very likely to continue even after mechanical agitation has ceased, limiting the quantitative reliability of ex situ studies. Examples of such transformations include continuation of the reaction, relaxation of activated phases, or reaction of mechanically activated material with air during sample preparation.

These obstacles can be circumvented by recently introduced techniques for in situ reaction monitoring, first developed using high energy synchrotron X-ray diffraction [97,99], and followed by the development of a more readily accessible Raman spectroscopy technique [215]. Most recently, a combined synchrotron X-ray diffraction and Raman monitoring system was presented by the Emmerling group [98]. The first in situ X-ray diffraction study of a mechanochemical reaction addressed the reactions of ZnO with different imidazole derivatives under neat grinding, LAG and ILAG conditions [97,216]. This study confirmed the appearance of low-density frameworks as intermediates in the formation of close packed ZIFs, not only for the ILAG reaction of ZnO and H**EtIm** but also, for example, for the reaction of ZnO with H**Im** in the presence of a limited amount of DMF: in situ monitoring revealed the initial formation of the open Zn(**Im**)_2_ framework with **cag** topology, which subsequently transformed into the close-packed *zni*-Zn(**Im**)_2_. For the reactions involving H**MeIm**, the time-dependent change in the intensity of diffraction lines of the ZIF-8 product revealed complete conversion within eight minutes for the ILAG reaction, whereas LAG led to maximum, incomplete conversion within 30 min. In both cases, product revealed sigmoidal behaviour, suggesting ZIF-8 formation from an initially amorphous product via an Avrami-type nucleation and growth mechanism.

**Figure 10:**
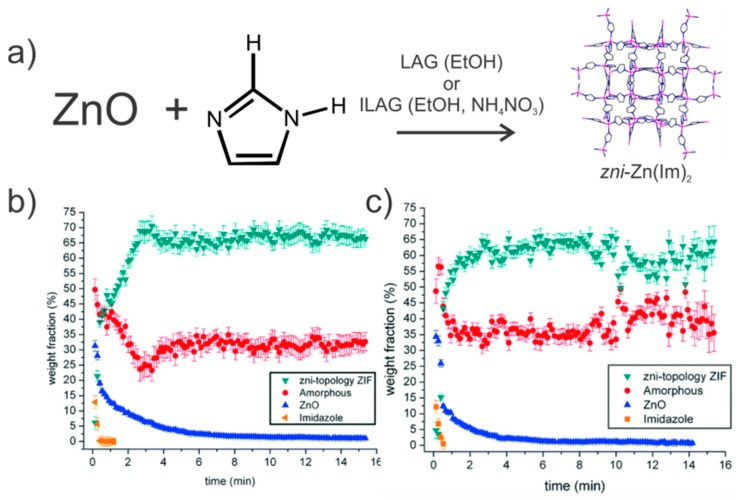
Quantitative in situ monitoring of the mechanochemical synthesis of the close-packed framework *zni*-Zn(**Im**)_2_: (**a**) the reaction scheme; (**b**) time-dependent change in weight fractions of the reactant, product, and amorphous phases in the LAG reaction, as determined by Rietveld analysis, and (**c**) time-dependent change in weight fractions of the reactant, product, and amorphous phases in the ILAG reaction, as determined by Rietveld analysis. Adapted with permission from [217].

Quantitative evaluation of the reaction kinetics was achieved by Halasz and co-workers by conducting LAG and ILAG reactions in the presence of an internal diffraction standard, revealing that the loss of ZnO diffraction lines over time resembles a 1st order reaction rate law [217]. This result is consistent with solution-type reaction rate laws observed by the James group using Raman spectroscopy [213], corroborating the pseudo-fluid model of a rapidly mixed mechanochemical reaction. Importantly, quantitative in situ studies enabled the evaluation of the amorphous content throughout the reaction. Rietveld analysis of in situ collected data indicated that the amorphous content during LAG and ILAG reactions achieves a steady state of ca. 30% by weight (Figure 10). Quantitative analysis of the reaction mixture ex situ, however, revealed no more than 7% by weight of amorphous material, indicating the rapid relaxation of amorphous materials during sample extraction and analysis.

**Figure 11:**
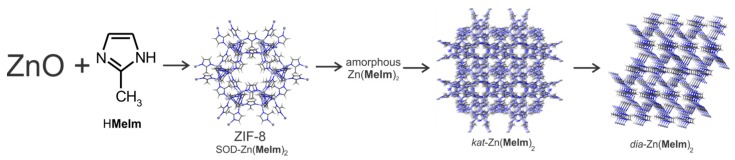
Scheme summarizing the sequence of SOD→*kat*→*dia* framework transformations in the mechanochemical reaction between ZnO and H**MeIm** in presence of dilute aqueous AcOH. In situ monitoring by X-ray diffraction led to the discovery of a topologically novel ZIF, *kat*-Zn(**MeIm**)_2_, which formed by crystallization of amorphous Zn(**MeIm**)_2_ and subsequently converted to the dense *dia*-Zn(**MeIm**)_2_ framework [218].

Reaction monitoring in situ led to unexpected discovery of a metastable polymorph of ZIF-8, exhibiting a previously not reported tetrahedral network topology named katsenite (three letter abbreviation *kat*) [218]. Specifically, attempts to conduct a greener synthesis of ZIF-8 by milling of ZnO and H**MeIm** with water as the milling liquid and acetic acid (AcOH) as the acid catalyst revealed the amorphisation of initially formed ZIF-8. Upon further milling, however, the amorphous material underwent crystallisation, first into the higher density *kat-*Zn(**MeIm**)_2_ and subsequently into the final, close-packed product *dia*-Zn(**MeIm**)_2_. The SOD→*kat*→*dia* sequence again follows the Ostwald rule of stages (Figure 11). This was confirmed also by density functional theory (DFT) evaluation of framework stability. For a more comprehensive overview of in situ techniques for mechanochemical reaction monitoring, we recommend a recent review [99].

## 5. Synthesis Assisted by Solvent Vapour

While mechanochemistry offers attractive advantages for the synthesis of coordination compounds, including almost complete elimination of solvents and ability to conduct reactions quickly and at room temperature, there is evidence that transformations involving acid-base reactions and exchange of coordination bonds can also take place, albeit slower, even without significant input of mechanical energy. Such reasoning has recently given birth to attractive, solvent-free and low-energy synthetic approaches wherein the transformations of well mixed reactant solids are facilitated by exposure to suitable environmental conditions. Despite a number of different names for such techniques, including vapour digestion, accelerated aging or vapour-assisted aging, their common facet is the use or an organic or inorganic (e.g., water, ammonia) vapours to enhance transformations of solid-state reactants, effectively exploiting molecular mobility at the gas-solid interface to achieve clean and low-energy synthesis of molecules and materials. Some of the early systematic studies of such reactivity has been reported in the pioneering quantitative studies of Byrn et al. on spontaneous reactivity in mixtures of carboxylic acid-based APIs (e.g., flufenamic acid) and the excipient MgO upon exposure to humid air [219,220]. While oriented primarily towards understanding of the stability of pharmaceutical formulations, this work clearly demonstrated how vapour-assisted reactivity permits room-temperature transformation of high-melting substances, such as the well-known refractory material MgO (melting point >2800 °C).

### 5.1. Vapour Digestion

The use of organic vapours as a catalyst to induce reactivity in mixtures of solids was first proposed as a solvent-free technique for the synthesis of hydrogen-bonded organometallic solids [221] by the Braga group, who exposed mixtures of a pyridine-substituted ferrocene and suberic acid to vapours of different organic liquids. Besides demonstrating a simple means to induce solid-state reactivity, this work also demonstrated a structure-directing effect of the organic vapours, as non-polar liquids led to the assembly of hydrogen-bonded cocrystals, and polar liquids led to a proton-transfer reaction to form a salt. Vapour digestion has since been extensively used in the synthesis of pharmaceutically relevant cocrystals, as well as in the assembly of metal-organic fluorescent and phosphorescent materials based on copper iodide clusters [123].

### 5.2. Accelerated Aging: Synthesis of MOFs Inspired by Mineral Neogenesis

In 2013, Qi and co-workers [222] demonstrated that exposure of static (i.e., not stirred) solid-state mixtures of oxalic acid with diverse main group and transition metal oxides to elevated humidity can enable the quantitative synthesis of metal oxalate coordination polymers. For example, no reaction was observed upon aging of a 1:1 stoichiometric mixture of zinc oxide and oxalic acid at room temperature and relative humidity (RH). However, exposure to high humidity (98% RH) at 45 °C led to quantitative formation of the 1-D chain polymer zinc oxalate hydrate.

Although the use of high humidity at mild conditions represents a novel direction in the synthesis of metal-organic materials, including MOFs, the transformations themselves are highly reminiscent of geological processes of mineral neogenesis (or mineral weathering) that take place upon exposure of simple binary minerals (e.g., metal oxides, sulphides, etc.) to small organic molecules of biological origin, such as oxalic acid that is excreted by lichens and is also abundant in guano, in the form of ammonium oxalate. Indeed, the formation of metal oxalate deposits through action of lichens of guano is a well-known geological phenomenon, leading to minerals such as Mooloite, Lindbergite, etc. [223,224].

**Figure 12:**
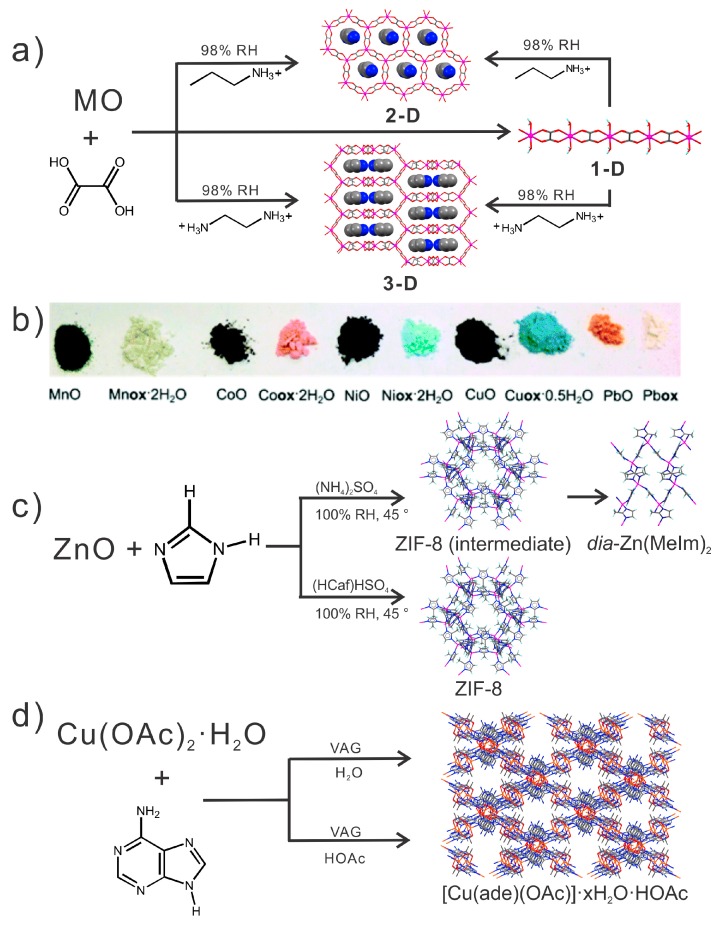
Versatility of accelerated aging and related techniques for the synthesis of MOFs and coordination polymers: (**a**) templated synthesis of 1-D, 2-D, and 3-D metal oxalate structures from metal oxides, or by solid-state reaction and rearrangement of a corresponding 1-D metal oxalate polymer [222]; (**b**) selected metal oxide reactants and corresponding metal oxalate products formed by aging in presence of oxalic acid. Adapted with permission from [222]; (**c**) Selective synthesis of ZIF-8 when using caffeinium sulfate (H**Caf**^+^)(HSO_4_^−^) as the accelerated aging catalyst, in contrast to the production of non-porous *dia*-Zn(**MeIm**)_2_ with catalytic (NH4)_2_SO_4_ additive [49,50]; (**d**) rapid synthesis of Cu-adeninate bio-MOF by vapour-assisted aging [225].

Importantly, the accelerated aging transformations could be readily directed towards the formation of open anionic MOF structures by addition of suitable organoammonium templating species in the static reaction mixtures (Figure 12a). Specifically, the addition of 1,3-propanediammonium oxalate induced the formation of 2-D metal oxalate MOFs with honeycomb (**hcb**, “chickenwire”) topology, whereas the addition of *n*-propylammonium oxalate induced the formation of open 3-D anionic MOFs [222]. These results are consistent with structure-templating studies in solution described by Cheetham and Rao [80]. In this way, accelerated aging enabled the synthesis of previously reported zinc MOFs directly from the oxide, as well as the synthesis of previously not known isostructural MOFs based on Ni(II) and Co(II) ions.

This study also established that different metal oxides exhibit different rates of conversion upon accelerated aging with oxalic acid. In most cases, reaction rates could be enhanced by brief milling of the solid-state mixture. With MnO, such mechanical activation also affected the nature of the final product, as enhanced reactivity led to the formation of less stable (kinetic) polymorph of manganese(II) oxalate dihydrate coordination polymer.

Differences in reactivity of different metal oxides were used for kinetic separation of solid mixtures. For example, exposing a 1:1:1 stoichiometric mixture of ZnO, CuO and oxalic acid to 98% RH and 45 °C led to selective transformation of ZnO into the hydrated zinc oxalate polymer, leaving behind CuO. The resulting mixture was readily resolved by flotation with a dense liquid, enabling a completely solvent-free separation of zinc and copper in their oxide form. Similar kinetic resolution of binary metal oxide mixtures was also demonstrated for systems comprising PbO and CuO, as well as ZnO and PbO, providing an attractive, clean methodology for resolution of mineral concentrates of base metals (Figure 12b) [222,226].

Accelerated aging is also readily applicable to materials not found in geological context, such as ZIFs [49,50]. So far, accelerated aging has enabled the spontaneous self-assembly of a range of ZIFs from metal oxides (CoO, ZnO) and 2-substituted imidazoles H**Im**, H**MeIm** and H**EtIm**. Although exposing solid-state mixtures of an oxide with an imidazole to high humidity (98%–100% RH) and mild temperature (45 °C) led to poor conversion or none at all, addition of catalytic amounts of protic salts was found to enable quantitative or nearly quantitative formation of ZIFs, without any bulk solvent. For example, a mixture of ZnO with H**Im** underwent partial conversion to form the non-porous *zni*-Zn(**Im**)_2_ framework. However, the addition of ca. 5 mol % (with respect to zinc) of NH_4_NO_3_ afforded complete conversion to the *zni*-topology framework product upon exposure to 100% RH and 45 °C. Similarly, reactions with H**MeIm** and H**EtIm** in the presence of a catalytic salt afforded non-porous diamondoid topology (*dia*) Zn(**MeIm**)_2_ and quartz topology (*qtz*) Zn(**EtIm**)_2_ MOFs [49].

**Figure 13:**
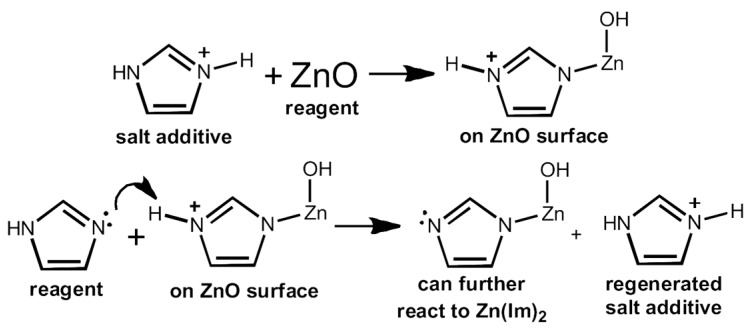
Proposed proton-transfer catalytic cycle in the accelerated aging synthesis of ZIFs, initiated by ammonium salts and perpetuated by imidazolium cations [49,50].

Reaction enhancement by protic salts was explained by an imidazolium-based proton transfer cycle. The aging reaction of (NH_4_)_2_SO_4_ and H**Im** revealed the formation of an imidazolium sulfate salt, (H_2_**Im**)_2_SO_4_·H_2_O, suggesting that accelerated aging reactions involve an imidazolium species that is sufficiently acidic to react with a metal oxide and is also regenerated throughout the reaction. In such a scenario, the reaction proceeds through the formation of a metal imidazole complex on the metal oxide surface, which is subsequently deprotonated by the remaining imidazole reagent to regenerate the active imidazolium species (Figure 13).

This proposed mechanism (Figure 13) enabled the targeted development of imidazolium and xanthine salt catalysts, resulting in a library of potential catalysts that can be used to screen for and optimise accelerated aging syntheses of MOFs. Such systematic investigation revealed caffeinium sulfate as a particularly successful catalyst for the synthesis of ZIF-8, which was obtained in gram amounts within a day. The prepared ZIF-8 exhibited a surface area on par to solvothermally-prepared material as well as commercially available Basolite Z1200^Ⓡ^ (Figure 12c) [50]. With H**EtIm**, the accelerated aging reaction was optimised to enable the first bulk synthesis of microporous RHO-Zn(**EtIm**)_2_. Importantly, a bulk solution-based synthesis of this ZIF was reported only later. Similarly, accelerated aging of CoO with H**Im**, H**MeIm** and H**EtIm** led to the quantitative formation of ZIFs with *zni*-, SOD-, and *qtz*-topologies, demonstrating the ability to obtain a diversity of open and close-packed metal-organic architectures from dense, normally inert starting materials in the solid state, without any solvent and under mild conditions [50].

A two-step synthetic procedure, that combines activation by milling, followed by aging, was developed by the Yuan group to achieve the conversion of CuO into the popular, commercially relevant framework HKUST-1 [227]. The procedure consists of ILAG of CuO and trimesic acid in the presence of DMF and NH_4_Cl salt additive. Exposure of the resulting solid to water vapour led to the assembly of the HKUST-1 framework within 6 h. Key to the aging formation of HKUST-1 appears to be the intermediate active species Cu(NH_3_)_2_Cl_2_ formed in the pre-milling step. The two-step procedure can also be performed if initial milling is conducted in the absence of a liquid phase, i.e., by milling in the presence of 5 mol % NH_4_Cl. However, in this case the quantitative assembly of HKUST-1 required significantly more time, 6 days [227].

Vapour-assisted solid-state reactivity was applied to the synthesis of a MOF based on a biologically relevant molecule (a Bio-MOF) by exposing equimolar mixtures of copper(II) acetate monohydrate and adenine (H**ade**) to humidity or acetic acid vapour (Figure 12d). The aging reaction, which yields the open framework Cu(**ade**)(**OAc**) in the form of a solvate with water and acetic acid, was accelerated if initial mixing of the reagents was conducted by brief ball milling, rather than by manual grinding. Most importantly, the use of acetic acid vapour was key to achieving rapid conversion of the reaction mixture. Exposure to humidity led to complete conversion after 12 h at room temperature, whereas AcOH vapours induced the quantitative assembly of the Bio-MOF in 30 min. In contrast, attempts to use MeOH vapours led to incomplete reaction that no longer advanced after 6 h [225].

An interesting demonstration of the potential of vapour-assisted reactivity in activating metal oxides involved exposure of mixtures of lanthanide metal oxides and trimesic acid to vapours of water or organic solvents (EtOH, DMF). After three days exposure to water vapour, oxides of lanthanum, neodymium, samarium and europium yielded a 1-D coordination polymer of composition M(**btc**)(H_2_O)_6_ (M = La, Nd, Sm, Eu) [227]. Similar reactivity was observed upon exposure to EtOH vapour, but with neodymium exhibiting only partial conversion. With DMF, the same product was obtained, but this time only in the case of lanthanum and europium. These results represent the first observation of solvent-free transformation of highly stable oxides of three-valent metals into metal-organic materials. The vapour-assisted aging reactivity of lanthanide metal carbonates led to the same product except, surprisingly, Tb_2_(CO_3_)_2_, which yielded the open framework Tb(**btc**)(H_2_O), exhibiting 6 Å-wide pores.

In addition to enabling the formation of MOFs from metal oxides, the vapour-assisted aging technique was shown to promote the interconversion of 1-D, 2-D, and 3-D structures [228]. For example, aging the 1-D coordination polymer Zn(**bdc**)(H_2_O)_2_ in DMF vapours afforded the formation of the porous 2-D framework Zn(**bdc**)(H_2_O)·DMF. Furthermore, the aging of Zn(**bdc**)(H_2_O)·DMF in MeOH vapour resulted in its conversion to the 3-D MOF Zn(**bdc**)(H_2_O). Both transformations were demonstrated to be reversible by exposing Zn(**bdc**)(H_2_O) and Zn(**bdc**)(H_2_O)·DMF to DMF and water vapour, respectively.

### 5.3. Geological Significance

**Figure 14:**
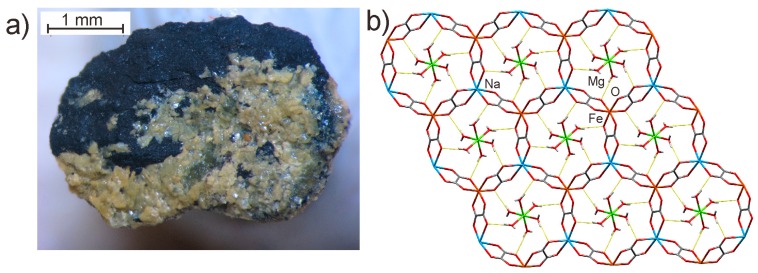
Minerals with MOF structures: (**a**) sample of stepanovite and (**b**) a top-down view of a single **hcb**-sheet in the crystal structure of stepanovite, with hydrogen bonds indicated by yellow dotted lines [229].

The targeted formation of 2-D and 3-D MOFs by templating the aging processes that in geological environments produce oxalate-based coordination polymers suggests that, under suitable conditions, mineral weathering could also lead to the formation of MOFs in nature. Indeed, this possibility is evidenced in the recent report of 2-D **hcb**-topology oxalate framework structures of the rare minerals zhemchuzhnikovite and stepanovite [229]. Both minerals were found to consist of layered MOF structures analogous to those found in magnetic materials designed by Decurtins group [230], or the proton-conducting materials developed by the Kitagawa group [231]. In stepanovite, the layers are arranged in ABCABC fashion and consist of octahedral Fe^3+^ and Na^+^ nodes bridged by oxalate anions (Figure 14a). In zhemchuzhnikovite, the Fe^3+^ nodes are partially replaced by Al^3+^ which also brings about a different, ABAB type of **hcb**-layer stacking. In each case, the framework pores are host to hydrated Mg(H_2_O)_6_^2+^ ions (Figure 14b).

Whereas network structures of known minerals have for long been a source of inspiration for the synthesis of coordination polymers and MOFs, as illustrated by the concept of mineralomimetic chemistry [232,233], zhemchuzhnikovite and stepanovite are first examples of known minerals that are, in fact, MOFs. Although the mechanisms of formation of zhemchuzhnikovite and stepanovite remain unknown, their open framework structures and the fact that metal oxalates in nature often form through mineral weathering, suggest that further studies of accelerated aging could be also of value for understanding geological processes leading to MOF minerals [229].

### 5.4. Accelerated Aging as a Materials Testing Procedure

Accelerated aging syntheses of open ZIFs reveal unexpected dynamics of the open metal-organic structures. For example, attempts to assemble an open MOF by aging of ZnO and H**MeIm** in the presence of simple ammonium salts revealed that the initially formed SOD-topology ZIF-8 structure readily collapses into the dense, thermodynamically more stable *dia*-Zn(**MeIm**)_2_. Similarly, in the analogous reaction with H**EtIm**, the initially obtained RHO-Zn(**EtIm**)_2_ rearranges into the non-porous *qtz*-Zn(**EtIm**)_2_ unless a suitable space-filling agent is present [49,50]. Whereas these synthetic obstacles could be resolved by using more advanced protic catalysts for accelerated aging reactions, they also suggest that aging in high humidity could be employed as a simple MOF stability testing procedure. Indeed, accelerated aging bears resemblance to standard procedures for testing the environmental stability of pharmaceutical solids, and exposure to high humidity was explored as a means to evaluate MOF stability by the Kaskel group [234] and the Zaworotko group [235].

**Figure 15:**
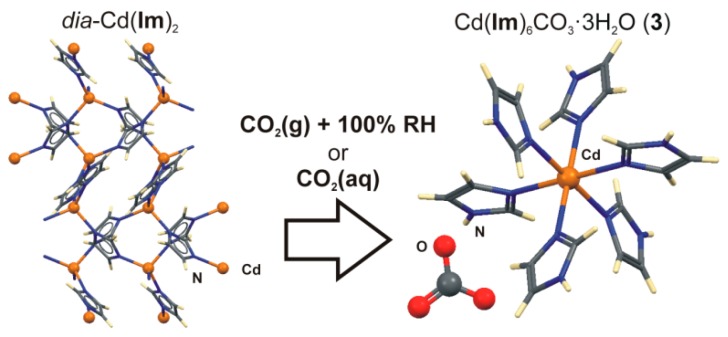
Degradation of the *dia*-topology framework Cd(**Im**)_2_ into the complex carbonate Cd(H**Im**)_6_CO_3_·3H_2_O by aging in humid carbon dioxide [236]. Adapted with permission from Reference [236].

Accelerated aging was used as a means to evaluate ZIF stability in complex atmospheres by exposing open and close-packed ZIFs of Zn, Co(II) and Cd to gaseous Ar, N_2_ or CO_2_ in the presence of water vapour [236]. All ZIFs were largely stable in humid Ar and N_2_. Humid CO_2_, however, induced unexpected and rapid transformation of almost all ZIFs, with the exception of *zni*-Zn(Im)_2_, into complex metal carbonates. Whereas the structures of these products remain unknown, the presence of imidazole and carbonate species was confirmed by ssNMR and ATR infrared analysis. For cadmium-based ZIFs, PXRD analysis revealed the formation of CdCO_3_ along with a complex carbonate based on Cd(HIm)_6_^2+^ cations (Figure 15). The sensitivity of ZIFs to moist CO_2_ contrasts their known stability in dry CO_2_ and resistance to steam or boiling water [237,238], highlighting the importance of using complex atmospheres to evaluate the stability of MOFs and their suitability for particular applications [239].

## 6. Thermochemical Solid-State Synthesis

The use of liquid additives or templating auxiliaries, although advantageous for previously discussed synthetic methods, can cause problems in the context of certain synthetic environments. For example, the production of lanthanide MOFs is often limited to oxygen-based coordination environments, due to the preference for binding oxygen. In addition, this behaviour can be inhibitory for the synthesis of lanthanide MOFs due to the tendency for the metal to preferentially bind coordinating solvents such as DMF. In that context, avoiding the use of solvents altogether, i.e., synthesis in the solid state, can be advantageous. To that end, the Müller-Buschbaum group devised the completely solvent-free synthesis of a europium(II)-based MOF comprising the ligand 1*H*-1,2,3-triazole (H**tz**) [240]. Europium metal and H**tz** were sealed in inert atmosphere and allowed to react in the melt to afford the close-packed structure Eu_3_(**tz**)_6_(H**tz**)_2_. The structure consisted of octahedrally-coordinated Eu^2+^ ions surrounded by solely nitrogen-atom donating ligands. Following their work with lanthanides, the same group adapted the thermally-assisted solvent-free methodology towards the synthesis of transition metal-based ZIFs in the melt, namely incorporating cobalt(II) ions. The synthesis of the close-packed ZIF of *zni* topology, Co(**Im**)_2_, was achieved via a stepwise and temperature-sensitive process. The reaction of cobalt metal and H**Im** under vacuum at the different temperatures, 150 °C, 210 °C, or 300 °C resulted in the synthesis of three chemically and structurally distinct cobalt(II) imidazolates of decreasing H**Im**:cobalt ratio, Co_3_(**Im**)_6_(H**Im**)_2_, Co_4_(**Im**)_8_(H**Im**), and *zni*-Co(**Im**)_2_. Interestingly, heating already formed Co_3_(**Im**)_6_(H**Im**)_2_ to 210 °C resulted in the loss of H**Im** to form Co_4_(**Im**)_8_(H**Im**), and heating the compound further to 225 °C yielded the homoleptic *zni*-Co(**Im**)_2_. Interestingly, the stepwise formation of coordination polymers of high ligand content at the start of the reaction when there is a large amount of ligand available for reaction is consistent with the mass action effect, as was seen in the LAG synthesis of zinc fumarate coordination polymers [241].

**Figure 16:**
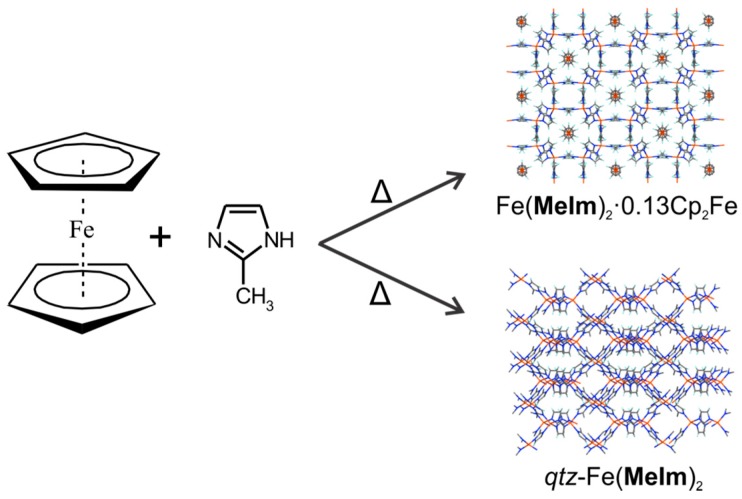
Thermally-assisted synthesis of iron(II)-based imidazolates [242,243].

Early contributions to the thermal synthesis of coordination polymers revealed the synthesis of iron imidazolates in the melt [242,243]. The Trotter group demonstrated that the reaction of ferrocene and H**Im** at 150 °C in a sealed tube under inert atmosphere yields the polymeric compound Fe_3_(**Im**)_6_(H**Im**)_2_. The methodology was also extended to the H**MeIm** ligand, which combined with Fe(II) to form the close-packed *qtz*-Fe(II)(**MeIm**)_2_ as well as an open framework incorporating ferrocene as a guest (Figure 16) [242,243]. While the work of Trotter and Müller-Buschbaum exemplified the fairly high reactivity between transition metals or lanthanides and imidazole ligands, Lin et al. demonstrated that the solid-state thermal synthesis could also be achieved from a simple metal oxide. Namely, the synthesis of MAF-4, also known as ZIF-8, was achieved by heating a solid mixture of ZnO and H**MeIm** for several hours [244]. Using higher temperatures resulted in greater crystallinity of MAF-4 material which was free of unreacted ligand and could be used for sorption experiments without subsequent activation by washing or evacuation. While such studied show that metal oxides are viable starting materials for the thermally-mediated synthesis of coordination polymers, the composition and topology of the products obtained were relatively uncontrollable. To circumvent this problem, the Román group presented one of the first examples of the structure-directed formation of MOFs in a solvent-free reaction. The synthesis of a series of zinc- and cobalt(II)-based imidazolate MOFs was achieved by means of the thermal reaction between metal oxides or hydroxides and azole ligands with or without the presence of templating agents [245]. Templating agents 1-butanol, **py**, and 4-methylpyridine (4**mpy**) enabled the formation of a series of topologically distinct structures incorporating the ligands H**Im** and H**MeIm**, including the novel frameworks *neb*-topology Zn(**Im**)_2_·(**py**)_0.5_ and *nog*-topology Co(**Im**)_2_·H_2_O)_2.5_·(4**mpy**)_0.2_. The Luque group expanded the scope of thermally-assisted synthesis of open coordination polymers from metal oxides and hydroxides by establishing the possibility to synthesize a series of zinc- and nickel(II)-based bio-MOFs based on adenine and carboxylic acid ligands [246].

## 7. Conclusions

We have attempted to provide a brief, but thorough overview of the most relevant concepts and recent advances in the use of mechanochemistry, as well as other solvent-free synthetic techniques for the assembly of extended structures based on coordination bonds. Of particular interest in that context is the synthesis of microporous MOFs, wherein solvent-free techniques have provided significant advantages over their more conventional solution-based or solvothermal counterparts. Indeed, the outstanding success of mechanochemistry and accelerated in providing access to a range of well-known and even industrially attractive microporous MOFs, without resorting to aggressive or toxic reagents, bulk solvent or elevated temperatures, provides a very strong impetus for the further development of these techniques. In that context, we believe that the recently developed methods for reaction monitoring will be of particular value, by providing not only the fundamental insight into reaction mechanisms required for rational development of mechanochemistry [247], and accelerated aging [248], but also by providing a means to efficiently screen the structural landscape accessible to different MOF compositions.

## Figures and Tables

**Table 1:** Independence of co-crystal formation between caffeine and L-tartaric acid on the solubilities of the reagents in selected LAG solvents [168].

Liquid Additive	Solubility		Product
	caf	L-ta	
none (neat grinding)	—	—	no reaction
water	15	400	**caf** hydrate
MeOH	10	170	cocrystal
EtOH	6	72	cocrystal
*i*-propanol	3	27	cocrystal
*n*-butanol	3	16	cocrystal
2,2,2-trifluoroethanol	450	2	cocrystal + **caf**
benzene	<2	<2	cocrystal + **caf**
fluorobenzene	15	<2	cocrystal + **caf**
acetonitrile	21	13	cocrystal
nitromethane	45	<2	cocrystal
chloroform	135	<2	cocrystal
ethyl acetate	7	9	cocrystal
di-*i*-propyl ether	<2	<2	cocrystal
ethylmethyl ketone	8	15	cocrystal
cyclohexane	<2	<2	no reaction

**Table 2:** Comparison of advantages and limitations of mechanochemical and solvothermal techniques for MOF synthesis.

**Solution or solvothermal synthesis**	**Advantages**
	• readily obtained single crystals
	• structural characterization by single crystal X-ray diffraction
	**Limitations**
	• requires soluble precursors
	• reagent dissolution requires heat and/or aggressive reagents (organic solvents, acids, bases)
	• large amounts of solvent waste generated
	• potentially hazardous handling of corrosive/explosive metal salts (chlorides, nitrates) in presence of organic liquids
	• reactions generate waste mineral acids or their salts (e.g., HNO_3_, HCl)
	• not applicable to products or reagents sensitive to heat or solvents
**Mechanochemistry by neat grinding**	**Advantages**
	• rapid synthesis (30–60 min) on multigram scale
	• quantitative yields when using metal salts as precursors
	• no reagent excess
	• no solvent waste
	• allows access to products and reactants sensitive to heat or solvents
	**Limitations**
	• structural characterization requires PXRD coupled with other techniques (e.g., solid-state NMR, thermal analysis, infrared spectroscopy)
	• neat milling may lead to unwanted product amorphization
**LAG mechanochemistry**	**Advantages**
	• rapid synthesis (30–60 min) on multigram scale
	• quantitative yields when using metal salts, carbonates and sometimes oxides
	• no solvent waste
	• no reagent excess
	• control of product topology by choice of liquid additive
	• allows access to products and reactants sensitive to heat or solvents
	• highly crystalline product
	**Limitations**
	• structural characterization requires PXRD coupled with other techniques (e.g., solid-state NMR, thermal analysis, infrared spectroscopy)
**ILAG mechanochemistry**	**Advantages**
	• rapid synthesis (30–60 min) on multigram scale
	• quantitative yields with metal salts, carbonates and oxides
	• no solvent waste
	• no reagent excess
	• control of product topology by choice of liquid or salt additives
	• allows access to products and reactants sensitive to heat or solvents
	• highly crystalline product
	**Limitations**
	• structural characterization requires PXRD coupled with other techniques (e.g., solid-state NMR, thermal analysis, infrared spectroscopy)
